# On the Six-Vertex Model’s Free Energy

**DOI:** 10.1007/s00220-022-04459-x

**Published:** 2022-09-06

**Authors:** Hugo Duminil-Copin, Karol Kajetan Kozlowski, Dmitry Krachun, Ioan Manolescu, Tatiana Tikhonovskaia

**Affiliations:** 1 Institut des Hautes Études Scientifiques, Bures-sur-Yvette, France; 2 Université Paris-Saclay, Gif-sur-Yvette, France; 3 Université de Genéve, Geneva, Switzerland; 4 ENS Lyon, Lyon, France; 5 University of Fribourg, Fribourg, Switzerland

## Abstract

In this paper, we provide new proofs of the existence and the condensation of Bethe roots for the Bethe Ansatz equation associated with the six-vertex model with periodic boundary conditions and an arbitrary density of up arrows (per line) in the regime $\Delta <1$. As an application, we provide a short, fully rigorous computation of the free energy of the six-vertex model on the torus, as well as an asymptotic expansion of the six-vertex partition functions when the density of up arrows approaches 1/2. This latter result is at the base of a number of recent results, in particular the rigorous proof of continuity/discontinuity of the phase transition of the random-cluster model, the localization/delocalization behaviour of the six-vertex height function when $a=b=1$ and $c\ge 1$, and the rotational invariance of the six-vertex model and the Fortuin–Kasteleyn percolation.

## Introduction

### Definition of the model

**Fig. 1:**
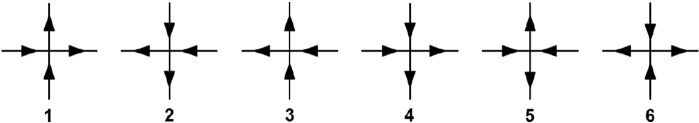
The 6 possibilities, or “types” of vertices in the six-vertex model. Each type comes with a weight $a_1,a_2,b_1, b_2, c_1,c_2$

The six-vertex model, first proposed by Pauling [36] in 1935 to study the residual entropy of ice, became the archetypical example of a planar integrable model with Lieb’s solution of the model in 1967 in its anti-ferroelectric and ferroelectric phases [29–32] using the Bethe Ansatz. We refer to [3, 33, 39] for detailed expositions and reviews and to [2] for the most general solution. The six-vertex model on the torus is defined as follows. For $N,M>0$ with *N* even, let ${{\mathbb {T}}}_{N,M} := ({\mathbb {Z}}/N{\mathbb {Z}})\times ({\mathbb {Z}}/M{\mathbb {Z}})$ be the *N* by *M* torus. An *arrow configuration* $\omega$ is a choice of orientation for every edge of ${\mathbb {T}}_{N,M}$. We say that $\omega$ satisfies the *ice rule* (or equivalently that it is a *six-vertex configuration*) if every vertex of ${\mathbb {T}}_{N,M}$ has two incoming and two outgoing edges in $\omega$. These edges can be arranged in six different types around each vertex as depicted in Fig. 1, hence the name of the model. One may easily check that the ice-rule guarantees that each horizontal line of vertical edges contains the same number of up arrows. From now on, let $\Omega ({\mathbb {T}}_{N,M})$ (resp. $\Omega ^{(n)}({\mathbb {T}}_{N,M})$) be the set of six-vertex configurations (resp. containing exactly *n* up arrows on each line).

For parameters $a_1,a_2, b_1,b_2, c_1,c_2\ge 0$, define the *weight* of a six-vertex configuration $\omega$ to be

$$\begin{aligned} W_\mathrm {6V}(\omega ) := a_1^{n_1}a_2^{n_2}b_1^{n_3} b_2^{n_4}c_1^{n_5} c_2^{n_6}, \end{aligned}$$

where $n_i$ is the number of vertices of ${\mathbb {T}}_{N,M}$ having type *i* in $\omega$. In this paper, we choose to focus on $a_1=a_2=a$, $b_1=b_2=b$ and $c_1=c_2=c$. Some of the results of this paper may extend to the asymmetric case and will be the object of a future paper.

Define the *partition functions* of the six-vertex model and of the six-vertex model with *n* up arrows per line, respectively, by setting

$$\begin{aligned} Z({\mathbb {T}}_{N,M},a,b,c)&:=\sum _{\omega \in \Omega ({\mathbb {T}}_{N,M})} W_\mathrm{6V}(\omega ),\\ Z^{(n)}({\mathbb {T}}_{N,M},a,b,c)&:=\sum _{\omega \in \Omega ^{(n)}({\mathbb {T}}_{N,M})} W_\mathrm{6V}(\omega ). \end{aligned}$$

In the analysis of the model, it is customary to introduce the parameter

1 $$\begin{aligned} \Delta :=\frac{a^2+b^2-c^2}{2ab}. \end{aligned}$$

**Fig. 2:**
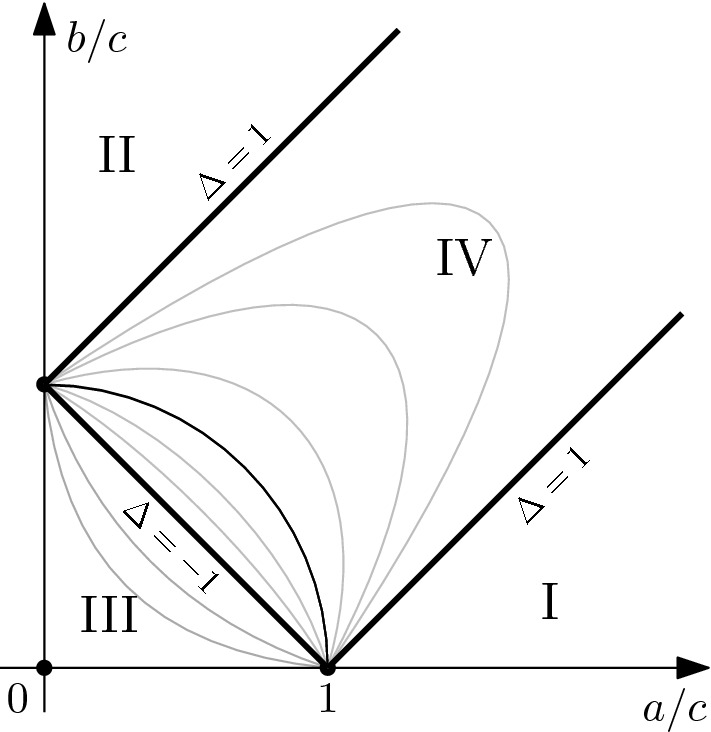
The expected phase diagram of the six-vertex model contains four regions: I and II are called ferroelectric, III is antiferroelectric and IV is disordered. The latter two regions correspond to $\Delta < -1$ and $\Delta \in [-1,1]$, respectively. The present paper concerns regions III and IV only. The gray lines represent lines of constant $\Delta$; the black quarter-circle corresponds to $\Delta = 0$

Below, we consider the region of parameters (*a*, *b*, *c*) such that $\Delta < 1$; see Fig. 2 for the phase diagram of the model.

### Main results for the symmetric six-vertex model

It appears convenient to adopt a parameterisation of the weights which makes transparent the connection with the algebraic Bethe Ansatz construction of the model’s transfer matrix. We thus introduce auxiliary parameters $\theta \in (0,\pi )$, $r\in {\mathbb {R}}_+$ and $\zeta$ such that^1^

- for $$-1< \Delta < 1 $$, $$\Delta =-\cos \zeta $$ with $$\zeta \in (0,\pi )$$,
  2 $$\begin{aligned} a\sin {\tfrac{\zeta }{2} } :=r\sin {(1-\tfrac{\theta }{\pi })\zeta }, \quad b\sin {\tfrac{\zeta }{2} }:=r\sin { \tfrac{\theta \zeta }{\pi } }, \quad c:=2r\cos { \tfrac{\zeta }{2} }, \end{aligned}$$
- for $$\Delta =- 1 $$,
  3 $$\begin{aligned} a := 2 r \tfrac{\pi -\theta }{\pi }, \quad b := 2 r \tfrac{\theta }{\pi }, \quad c:=2r, \end{aligned}$$
- for $$ \Delta < -1 $$, $$\Delta =-\cosh \zeta $$ with $$\zeta \in {\mathbb {R}}_+$$,
  4 $$\begin{aligned} a\sinh {\tfrac{\zeta }{2} } :=r\sinh {(1-\tfrac{\theta }{\pi })\zeta }, \quad b\sinh { \tfrac{\zeta }{2} }:=r\sinh { \tfrac{\theta \zeta }{\pi } }, \quad c:=2r\cosh { \tfrac{\zeta }{2} }. \end{aligned}$$

The first result goes back to Lieb [30–32] and Sutherland [38] and deals with the per-site *free energy* defined by

5 $$\begin{aligned} f(a,b,c):=\lim _{N\rightarrow \infty }\lim _{M\rightarrow \infty }\tfrac{1}{MN}\log Z({\mathbb {T}}_{M,N},a,b,c), \end{aligned}$$

in which the limits may be taken in any order as established in [33]. The mentioned papers characterised the per-site free energy relying on the same strategy as the original paper [41] which deals with the XXZ quantum spin chain. At the time, the closed expressions for *f*(*a*, *b*, *c*) were derived under the hypothesis of the so-called condensation of Bethe roots. As will be discussed more precisely later on in the introduction, the condensation property has nowadays been rigorously established. Here, we develop an alternative technique for proving condensation which, on the one hand, turns out to be particularly effective for our goals and, on the other hand, allows one to go beyond what can be rigorously proven within the existing scope of techniques.

#### Theorem 1

For every $a\ge b> 0$ and $c\ge 0$ such that $\Delta <1$ (*c.f.* (1)), using the parameterisation (2)–(4),6In particular $f(1,1,1)=\tfrac{3}{2}\log (\tfrac{4}{3})$ and $f(1,1,2)=2\log [2\Gamma (\tfrac{5}{4})/\Gamma (\tfrac{3}{4})]$, with $\Gamma$ the gamma function.

The value of *f*(1, 1, 1) was first obtained by Lieb and corresponds to the residual entropy of square ice.

Our second result deals with the following extension of the per-site free energy to generic values of *n* and *N*:

$$\begin{aligned} f_N^{(n)}(a,b,c):=\lim _{M\rightarrow \infty }\tfrac{1}{NM}\log Z^{(n)}({\mathbb {T}}_{N,M},a,b,c). \end{aligned}$$

It provides a characterisation of the subleading corrections to $f_N^{(n)}(a,b,c)$ as $n, N \rightarrow + \infty$ in such a way that $n/N \rightarrow 1/2$. The condition on *n* and *N* appearing in the statement below is technical and takes its origin in the statements of the subsequent theorems in this paper.

#### Theorem 2

For $N \ge 2$ even and $a,b,c\ge 0$ leading to $\Delta < 1$ (*c.f.* (1)), there exist constants $C,C(\zeta ),C'(\zeta ,\theta )\in (0,\infty )$ such that for every

7 $$\begin{aligned} n\le \tfrac{1}{2}N-C\min \{\zeta ^{-2},\log (N)^2\}, \end{aligned}$$

using the parametrisation (2)–(4), we have

8 $$\begin{aligned} f_N^{(n)}(a,b,c)&=f(a,b,c)+O(\tfrac{1}{N})\nonumber \\&\quad -(1+o(1)){\left\{ \begin{array}{ll}C(\zeta )\sin {\theta }\big (1-\tfrac{2n}{N}\big )^2&{}\hbox { if } -1\le \Delta< 1,\\ C'(\zeta ,\theta )(1-\tfrac{2n}{N})&{}\hbox { if } \Delta <-1,\end{array}\right. }\end{aligned}$$

where *o*(1) means a quantity tending to 0 as *n*/*N* tends to 1/2.

Notice that for $\Delta \in [-1,1)$, (8) only gives meaningful information when $\frac{N}{2} - n$ exceeds $\sqrt{N}$.

This extension has important applications for the six-vertex model and other related models. The six-vertex model lies at the crossroads of a vast family of two-dimensional lattice models; for instance, it has been related to the dimer model, the Ising and Potts models, Fortuin–Kasteleyn (FK) percolation, the loop *O*(*n*) models, the Ashkin–Teller models, random permutations, stochastic growth models, quantum spin chains, to cite but a few examples. Among such links, one can use the Baxter-Kelland-Wu mapping between the six-vertex model and FK percolation [4] to deduce from Theorem 2 and the dichotomy result of [13] that the phase transition of FK percolation on the square lattice is continuous if the cluster weight *q* satisfies $1\le q\le 4$, and is discontinuous for $q>4$. We refer to the papers where the results were proved (using alternative methods) for additional details [13, 14]. It should be mentioned that the continuity result of [13] may be deduced directly from Theorem 2 using the same procedure as in [18]. In the same spirit, the results can be used to derive dimerisation properties of the anti-ferromagnetic Heisenberg chain [1].

A second application of our results is related to the height function *h* of the six-vertex model, which can be proved to be localised (meaning that the variance of $h(x)-h(y)$ is bounded uniformly in $|x-y|$) whenever $a=b=1$ and $c>2$, and delocalised (meaning that the variance of $h(x)-h(y)$ tends to infinity logarithmically fast in $|x-y|$) when $a=b=1$ and $1\le c\le 2$. We refer to [16, 18, 21] for more details. It is conjectured more generally that the height-function is localised when $\Delta < -1$, and delocalised when $-1\le \Delta <1$. This property is closely related to the existence of a massive ($\Delta < -1$) and massless ($-1 \le \Delta < 1$) regime in the XXZ spin-1/2 Heisenberg chain; there, the ground state correlation functions of local operators at distance *m* decay exponentially fast in $m \rightarrow + \infty$ in the massive regime and algebraically in *m* in the massless regime. Indeed, one can show that the XXZ spin-1/2 Heisenberg Hamiltonian ground state generating function of the longitudinal spin-spin correlations does coincide with the generating function of variances of the height function of the six vertex model. Thus, the power-law decay of the correlators in the XXZ chain translates to the logarithmic growth of the variance of the height function for the six-vertex model.

Finally, another use of our results is in [17], where a refined version of Theorem 2 (see Sect. 8) is employed to show that the correlations of the height function of the six-vertex model are invariant under rotations in the scaling limit, when $a=b=1$ and $\sqrt{3}\le c\le 2$. This rotation invariance should in fact hold for every $c\in [1,2]$ (but this has not been proven yet) and be wrong for $c>2$ (when the height function localises, as discussed above). The argument of [17] involves the FK percolation representation of the six-vertex model, and the rotational invariance result also applies to critical FK percolation on ${\mathbb {Z}}^2$ with cluster weight $q \in [1,4]$.

### Transfer matrix of the six-vertex model and the Bethe Ansatz

In order to understand the large scale asymptotics of $Z^{(n)}({\mathbb {T}}_{N,M},a,b, c)$ with $0\le n\le N/2$, one introduces the *transfer matrix* $V_N=V_N(a,b,c)$ (that we do not write explicitly here; see e.g. [7]) defined as an endomorphism of the $2^N$-dimensional real vector space spanned by the basis $\big \{\mathbf \Psi _{\mathbf {x}}\big \}_{\mathbf {x}}$, where $\mathbf {x}=(x_1,\dots ,x_n)$ with $1\le x_1<\dots <x_n\le N$, $0\le n\le N$ (below, we use $|\mathbf {x}|:=n$ for the *length* of $\mathbf {x}$) and $\mathbf {\Psi }_{\mathbf {x}}=(\Psi _{\mathbf {x}}(1),\dots , \Psi _{\mathbf {x}}(N) )\in \{\pm 1\}^{N}$ is given by

$$\begin{aligned} \Psi _{\mathbf {x}}(i) := {\left\{ \begin{array}{ll} +1 \quad \hbox { if } i \in \{x_1,\dots , x_{|\mathbf {x}|}\},\\ -1 \quad \hbox { if } i \notin \{x_1,\dots , x_{|\mathbf {x}|}\}. \end{array}\right. } \end{aligned}$$

In particular, one finds that

9 $$\begin{aligned} Z({\mathbb {T}}_{N,M},a,b, c)&=\mathrm {Trace}[V_N(a,b,c)^M],\nonumber \\ Z^{(n)}({\mathbb {T}}_{N,M},a,b, c)&=\mathrm {Trace}[V_N^{(n)}(a,b,c)^M], \end{aligned}$$

where $V_N^{(n)}(a,b,c)$ is the block of the matrix $V_N(a,b,c)$ restricted to the vector space spanned by the $\mathbf {\Psi }_{\mathbf {x}}$ with $|\mathbf {x}|=n$. This vector space is indeed stable by $V_N(a,b,c)$ because of the conservation of the number of up arrow per horizontal line. In light of the above displayed equation, we have a clear interest in studying the spectral properties of $V_N(a,b,c)$ and $V_N^{(n)}(a,b,c)$. Standard arguments of rigorous statistical mechanics, see e.g. [33], allow one to conclude that

$$\begin{aligned} f(a,b,c) \, = \, \lim _{N\rightarrow + \infty } \tfrac{1}{N} \log \Lambda _{N}(a,b,c) \qquad \text {and}\qquad f_N^{(n)}(a,b,c)= \tfrac{1}{N} \log \Lambda _{N}^{(n)}(a,b,c), \end{aligned}$$

where $\Lambda _{N}(a,b,c)$ and $\Lambda _{N}^{(n)}(a,b,c)$ are the largest eigenvalues of $V_N(a,b,c)$ and $V_N^{(n)}(a,b,c)$, respectively. Note that since $V_N^{(n)}(a,b,c)$ is a Perron-Frobenius matrix, *c.f.* e.g. [31], $\Lambda _{N}^{(n)}(a,b,c)$ is the Perron-Frobenius eigenvalue of $V_N^{(n)}(a,b,c)$. The full transfer matrix $V_N(a,b,c)$ is not Perron-Frobenius, but it may be shown that its single largest eigenvalue is $\Lambda _{N}^{(0)}(a,b,c)$.

The coordinate Bethe Ansatz, introduced by Bethe [6] in 1931, provides mathematicians and physicists with a powerful way of obtaining eigenvalues of one-dimensional quantum models and of the transfer matrices of certain two-dimensional lattice models. In particular, Orbach [35] put it in a form allowing one to study the eigenvalues of the XXZ spin-1/2 Heisenberg chain, a model sharing the same eigenvectors as the six-vertex transfer matrix, *see* [34] for the explanation of this last fact. Further, since the visionary work of the Leningrad School [19], the coordinate Bethe Ansatz has been put into a fully algebraic framework, called nowadays the algebraic Bethe Ansatz, which is deeply connected with the representation theory of quantum groups. This picture strongly simplified the analysis of integrable models.

We now summarise the program corresponding to the implementation of the Bethe Ansatz to understand the asymptotics of the largest eigenvalue of $V_N^{(n)}(a,b,c)$. The survey [15] contains an elementary derivation of Bethe’s Ansatz intended for probabilists, and is a useful reference for most of what is discussed above.
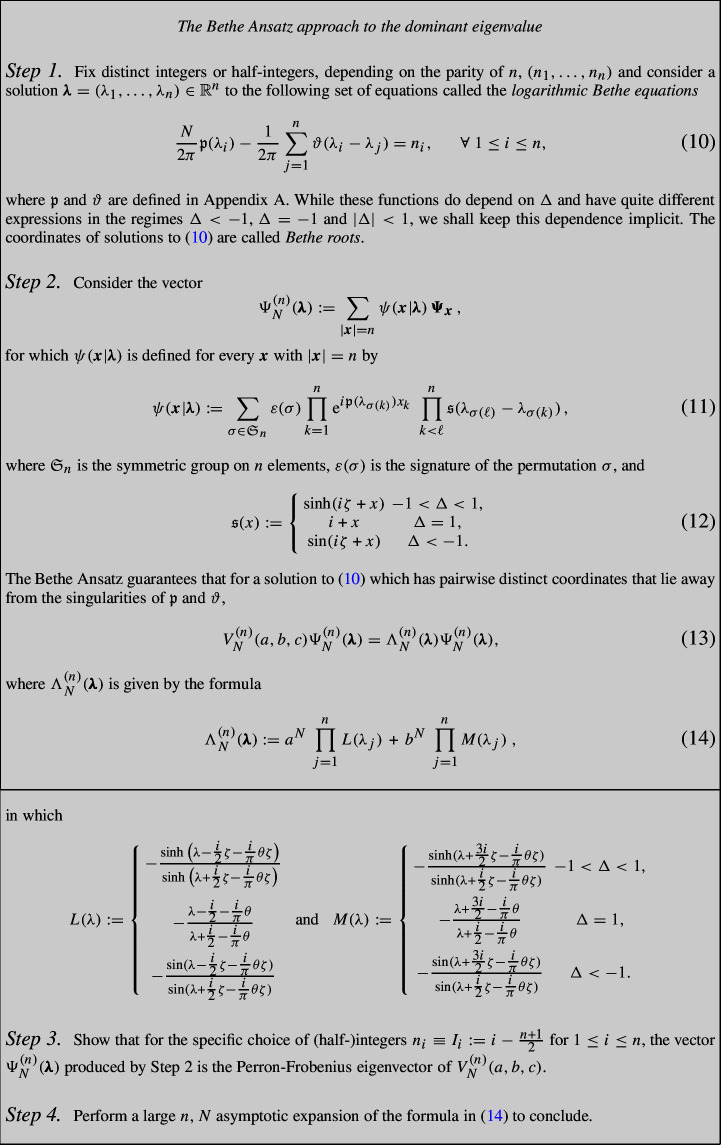


Note that the Bethe equations ensure that the Bethe roots are not poles of *L* or *M*, so that $\Lambda _N^{(n)}(\pmb \lambda )$ is indeed well defined. Also notice that the Bethe equations and the resulting vector $\Psi _N^{(n)}$ only depend on $\Delta$ (or equivalently on $\zeta$); the only dependence on *a* and *b* (or equivalently on $\theta$) is in the formula for $\Lambda _N^{(n)}(\pmb \lambda )$.

At this stage, implementing the above program rigorously requires particular attention at certain points, namely:

In Step 1, for a given choice of $\pmb n= (n_1,\dots , n_n)$, one must prove the existence of solutions to (10). In the regime $\Delta < 1$, Yang and Yang [41] proved the existence of Bethe roots when $n_i=I_i$ as above. Then Griffiths [22] established the existence of solutions to a certain class of (half-)integers $\pmb n$. More recently, Kozlowski [28] established the existence of solutions, as well as their uniqueness when *N* is large enough, for a wide class of (half-)integers $\pmb n$ describing the so-called particle-hole excitations.

In Step 2, in order to conclude from (13) that $\Lambda _N^{(n)}(\pmb \lambda )$ is an eigenvalue of $V_N^{(n)}(a,b,c)$, it must be shown that the Bethe roots’ coordinates are pairwise distinct and that $\Psi _N^{(n)}(\pmb \lambda )$ is non-zero. This was shown to hold for the solution $\pmb \lambda$ associated with $n_i=I_i$ by Yang and Yang [41]. For solutions having pairwise distinct coordinates that are associated with other choices of (half-)integers $\pmb n$ and which satisfy some form of condensation, *c.f.* later on, the non-vanishing of $\Psi _N^{(n)}(\pmb \lambda )$ for *N* large enough may be proved using the determinant representation for the norm of $\Psi _N^{(n)}(\pmb \lambda )$, which was conjectured in [20, 27] and rigorously proven in [26, 37].

In Step 3, one should argue that the vector $\Psi _N^{(n)}(\pmb \lambda )$ obtained using the specific choice of (half-)integers $I_i$ is indeed the Perron-Frobenius eigenvector of $V_N^{(n)}(a,b,c)$. This was first conjectured by Hulthén [25] and was established by Yang and Yang [41]. Checking that $\Psi _N^{(n)}(\pmb \lambda )$ is the Perron-Frobenius eigenvector is reasonably simple for $\Delta$ equal to 0 or $-\infty$ (for $\Delta =-\infty$ and general *n* this actually does require some effort). In order to extend the result to an interval of values of $\Delta$, one may prove the continuity or analyticity of $\Psi _N^{(n)}(\pmb \lambda )$ as a function of $\Delta$. If continuity is used, then one should additionally prove that $\Psi _N^{(n)}(\pmb \lambda )$ does not vanish outside of a discrete set of values of $\Delta$.

In Step 4, in order to perform the asymptotic expansion, one needs to prove some form of *condensation* of the Bethe roots $\pmb \lambda$, i.e. the convergence of the point measure $\texttt {L}_N^{(\pmb \lambda )}=\tfrac{1}{N} \sum _{a=1}^{n} \delta _{\lambda _a}$ towards a given measure in the large *N* limit. To be more precise, we should first introduce the *continuum Bethe equation* whose solution allows one to characterise the limiting measure. For $q \in [0,\infty ]$ when $|\Delta | \le 1$ and $q \in [0,\pi /2]$ when $\Delta <-1$, define $\rho (\cdot |q)$ as the solution (the unique solvability was thoroughly discussed, by different methods, in [12, 28, 42]) to the linear integral equation

15 $$\begin{aligned}&\rho (\lambda | q) + \int _{-q}^q K(\lambda -\mu ) \rho (\mu | q)d\mu = \xi (\lambda ),\qquad \forall \lambda \in {\mathbb {R}}, \end{aligned}$$

with $$K:=\tfrac{1}{2\pi }\vartheta '$$ and $$\xi :=\tfrac{1}{2\pi }{\mathfrak {p}}'$$.

When $n/N \rightarrow m \in [0,1/2]$ as $n,N\rightarrow +\infty$, the point measure $\texttt {L}_N^{(\pmb \lambda )}$ associated with the solution $\pmb {\lambda }$ to (10) corresponding to the choice of (half-)integers $n_i=I_i$ converges weakly towards ${\rho \big ( \lambda | Q(m) \big ) \pmb {\mathbb {1}}_{[-Q(m);Q(m)]}(\lambda ) d \lambda }$, in which *Q*(*m*) is the unique solution to

16 $$\begin{aligned} \int \limits _{ - Q(m) }^{ Q(m) } \rho ( \lambda |Q(m) ) d\lambda = m. \end{aligned}$$

The existence and uniqueness of *Q*(*m*) has been first proven in [12]. We also refer to “Appendixes B and D” for a proof of *Q*(*m*)’s existence. The uniqueness of *Q*(*m*) may be obtained as a consequence of Theorem 3 below, and will be discussed thereafter. For future reference, it may be useful to note that *Q* is increasing and $Q(1/2) = \pi /2$ when $\Delta < -1$ and $Q(1/2) = \infty$ when $|\Delta | \le 1$.

Condensation of Bethe roots was first proven when $0< \Delta <1$ by Gusev [24] for any *m* using convex analysis tools. Much later, Dorlas and Samsonov [10] used different convex analysis techniques to prove the same result and were also able to prove condensation for any *m* and $\Delta <-\Delta _0$ with $\Delta _0$ large enough, *viz*. perturbatively around $\Delta =-\infty$. More recently, Kozlowski [28] proved condensation for any value of $m\in [0,1/2]$ and $\Delta \in (-\infty ,1)$, in particular away from the region where convexity or perturbative arguments are applicable. That proof relied on developing a rigorous approach to dealing with the non-linear integral equations governing the so-called counting function of the Bethe roots that were introduced and handled, on a loose level of rigour in [5, 8, 40]. The non-linear integral equation method allowed to rigorously establish the condensation of Bethe roots associated with a large class of (half-)integers in (10), not only $n_i=I_i$, as well as to go beyond the limiting value, and to compute an all order asymptotic expansion in *N* for $\int {}{} f(\mu ) d \texttt {L}_N^{(\pmb \lambda )}(\mu )$ for any $\Delta <-1$ and $m \in [0,1/2]$, as well as for any $-1\le \Delta <1$ and $m \in [0,1/2)$. However, owing to the lack of certain compactness properties, the non-linear integral equation method does not allow one to reach rigorously^2^ an estimate beyond $\text {o}(1)$ for

17 $$\begin{aligned} \int {}{} f(\mu ) d \texttt {L}_N^{(\pmb \lambda )}(\mu ) \, -\int \limits _{-Q(\frac{n}{N})}^{Q(\frac{n}{N})} f(\mu ) \, \rho (\mu | Q(\tfrac{n}{N}) )d\mu \end{aligned}$$

when $$-1\le \Delta <1$$ and $$m=1/2$$.

In this work, we develop a method which allows one to estimate (17) up to a *O*(1/*N*) for $-1< \Delta <1$ and $m=1/2$ and up to a $O( \ln N/N)$ for $\Delta =-1$ and $m=1/2$. Reaching these values of the parameters in the model plays a very important role for the results obtained in [16–18] and this stresses the significance of our result.

### Results for Bethe’s equations

For $n \le N/2$, we will henceforth always consider the sequence of (half)-integers

18 $$\begin{aligned} n_i\equiv I_i:=i-\tfrac{n+1}{2} \qquad 1 \le i \le n, \end{aligned}$$

appearing in (10).

For $\Delta < 1$, recall that we are interested in the solutions $\pmb \lambda =(\lambda _1,\dots , \lambda _n) \in {\mathbb {R}}^n$ to

19 $$\begin{aligned} \frac{N}{2\pi }{\mathfrak {p}}(\lambda _i) - \frac{1}{2\pi } \sum _{j = 1}^n \vartheta (\lambda _i - \lambda _j) = I_i,\qquad \forall 1\le i\le n, \end{aligned}$$

where ${\mathfrak {p}}$ and $\vartheta$ are defined in “Appendix A”. We will also require that solutions are

- *symmetric*, meaning that $\lambda _{n+1-i}=-\lambda _i$ for every $1\le i\le n$,
- *strictly ordered*, meaning $\lambda _i<\lambda _{i+1}$ for every $1\le i<n$.

The first main result of this section is the existence of solutions to (19) without any assumption on $n\le N/2$ or $\Delta \ne -1$, with a quantitative control on how condensed these solutions are.

#### Theorem 3

(Existence of condensed solutions to discrete Bethe equations when $\Delta \ne -1$). There exists a constant $C>0$ such that for every $n\le N/2$ and every $\Delta \in (-\infty ,-1)\cup (-1,1)$, there exists a symmetric strictly ordered solution $\pmb \lambda =(\lambda _1,\dots , \lambda _n)$ to (19), which satisfies

20 $$\begin{aligned} \Big |\frac{1}{N} \sum _{j = 1}^n f(\lambda _j) - \int _{-{\mathfrak {q}} }^{ {\mathfrak {q}} } f(\lambda ) \rho (\lambda | {\mathfrak {q}} )\, d\lambda \Big | \le \frac{C}{\zeta N}\,\Vert f' \Vert _{L^1({\mathbb {R}})} \end{aligned}$$

for every $f:{\mathbb {R}} \rightarrow {\mathbb {R}}$ with integrable derivative. Above, $\zeta$ is related to $\Delta$ as in (2)–(4), and we introduced the shorthand notation

21 $$\begin{aligned} {\mathfrak {q}} \, := \, Q\big ( \tfrac{n}{N}\big ) . \end{aligned}$$

A solution satisfying (20) will be referred to as *condensed*. Note that the condensation is fairly quantitative but that the control degenerates when $\Delta$ is approaching $-1$. We refer to Theorem 6 below for the treatment of the case $\Delta =-1$.

The second theorem will be devoted to the existence of an analytic family of such solutions. The existence of a continuous family of solutions has been previously proven in [41]. Yet, we could not identify any use of the continuity property which warrants mentioning this stronger statement. On the contrary, a property that seems crucial for applications to Bethe’s Ansatz is the property of analyticity in $\Delta$ of the Bethe roots. Analyticity may also be directly inferred from the results of [28] for $\Delta <-1$ and all *m* as well as for $-1 \le \Delta <1$ and $m\in [0,1/2)$. In this paper, we extend these analyticity results (by another range of arguments) up to $m=1/2$ in the sense described by Theorem 4 below.

#### Theorem 4

(Analytic family of solutions to discrete Bethe equations). For every $\Delta _0<1$, there exist $N_0(\Delta _0)<\infty$ and $C_0(\Delta _0)<\infty$ such that there exists a *unique* family of condensed symmetric strictly ordered solutions $\Delta \mapsto \pmb \lambda (\Delta )$ to (19), which is analytic as a function of $\Delta$ on the following intervals:

- If $\Delta _{0}>-1$, on $(\Delta _0,1)$ as soon as $N\ge N_0(\Delta _0)$ and $n\le N/2-C_0(\Delta _0)$.
- If $\Delta _{0}<-1$, on $(-\infty ,\Delta _0)$ as soon as $N\ge N_0(\Delta _0)$ and $n\le N/2$.

Moreover, there exists $\Delta _0\in (-1,0)$ such that $N_0(\Delta _0)$ and $C_0(\Delta _0)$ can be taken to be 0.

We are currently unable to prove, with our method, the existence of an analytic solution for arbitrary $n\le N/2$ over the whole intervals $(-\infty ,-1)$ and $(-1,1)$. We refer to the remarks in Sect. 3.2 for more details. However, this fact appears to be closely related to the expected property that the model undergoes a phase transition of infinite order at $\Delta =-1$.

Our next result states that the eigenvalue (14) obtained from the Bethe roots provided by Theorem 4 is indeed the Perron-Frobenius eigenvalue of $V_N^{(n)}(a,b,c)$.

#### Theorem 5

(The Bethe Ansatz gives the Perron-Frobenius eigenvalue). Fix $n \le N/2$. For the analytic family of solutions $\Delta \mapsto \pmb \lambda (\Delta )$ on (*u*, *v*) to (19) given by Theorem 4 (with $(u,v) = (-\infty ,\Delta _0)$ or $(\Delta _0,1)$), the quantity $\Lambda _N^{(n)}\big (\pmb \lambda (\Delta )\big )$ constructed by (14) from $\pmb \lambda (\Delta )$ is the Perron-Frobenius eigenvalue of $V_N^{(n)}(a,b,c)$ for every *a*, *b*, *c* such that $\Delta \in (u,v)$.

The last two theorems have the following direct consequence. For $\Delta \ne -1$ and $n\le N/2$, consider the solution $\pmb \lambda (\Delta )$ provided by Theorem 4. Since the functions $\log | L(x)|$ and $\log | M(x)|$ are differentiable, the condensation and symmetry imply that

$$\begin{aligned} \tfrac{1}{N} \sum \limits _{j=1}^n \log |L\big (\lambda _j\big )| =\int \limits _{- {\mathfrak {q}} }^{ {\mathfrak {q}} } \log |L(\lambda )|\,\rho (\lambda |{\mathfrak {q}})d\lambda +O(\tfrac{1}{N}), \end{aligned}$$

and a similar expression for *M*. When $a>b$, one may check that the contribution to $\Lambda _N^{(n)}\big ( \pmb \lambda (\Delta ) \big )$ issuing from the *L* term is larger than the one issuing from the *M* term. This allows one to deduce from the transfer matrix formalism and Theorem 5 that^3^

22 $$\begin{aligned} f_N^{(n)}(a,b,c)&=\lim _{M\rightarrow \infty }\tfrac{1}{NM}\log \mathrm{trace}[V_N^{(n)}(a,b,c)^M]\nonumber \\&=\tfrac{1}{N} \log \big [ \Lambda _N^{(n)}\big ( \pmb \lambda (\Delta ) \big ) \big ] \nonumber \\&=\log a+\int \limits _{-{\mathfrak {q}} }^{ {\mathfrak {q}} } \log |L(\lambda )|\,\rho (\lambda |{\mathfrak {q}}) d\lambda + O(\tfrac{1}{N}) \end{aligned}$$

as long as $n,N,\Delta$ are in one the cases where Theorem 4 holds and $a \ge b$.

Theorems 1 and 2 follow from (22) once one can estimate efficiently the right-hand side. At the core of this estimate is the following observation going back to [42]. Let ${\mathcal {K}}$ be the operator acting on $L^2(I)$, where $I={\mathbb {R}}$ for $|\Delta |\le 1$ and $I=[-\tfrac{\pi }{2},\tfrac{\pi }{2}]$ for $\Delta < -1$, constructed from the integral kernel $K(\lambda -\mu )$; let ${\mathcal {R}}$ be defined by $(\mathrm {id}-{\mathcal {R}}) = (\mathrm {id}+{\mathcal {K}})^{-1}$. We refer to ${\mathcal {R}}$ as the resolvent, and to its integral kernel *R* as the *resolvent kernel*. Then, (15) is equivalent to the linear integral equation

23 $$\begin{aligned} \rho (\lambda |q)-\int _{I{\setminus }[-q,q]}R(\lambda -\mu )\rho (\mu |q)d\mu =\rho (\lambda ) \qquad \hbox { for all } \lambda \in {\mathbb {R}}, \end{aligned}$$

where $\rho = (\mathrm {id} - {\mathcal {R}})\xi$. The resolvent kernel *R* and $\rho$ are best expressed through their Fourier transforms/coefficients^4^

$$\begin{aligned} {\widehat{R}}:= \frac{{\widehat{K}}}{1+{\widehat{K}}} \quad \text {and} \quad {\widehat{\rho }}:= \frac{{\widehat{\xi }}}{1+{\widehat{K}}} . \end{aligned}$$

We refer to “Appendix A” for the explicit formulae.

Due to the definition of *Q*, we have that $I = [-Q(1/2),Q(1/2)]$, and thus $\rho (\lambda )=\rho \big ( \lambda | Q(\tfrac{1}{2}) \big )$. The rewriting of (15) as (23) has the advantage of putting emphasis on the perturbative structure of the equation for *q* located in the vicinity of $Q(\tfrac{1}{2})$.

Up to now, our results were always stated for $\Delta$ belonging to strict subintervals of $(-\infty ,-1)$ or $(-1,1)$. We conclude this section with a result dealing with the case $\Delta =-1$.

#### Theorem 6

There exist $N_0,C_0,C_1>0$ such that for every $N\ge N_0$ and

$$\begin{aligned} n\le \frac{N}{2}-C_1(\log N)^2,\end{aligned}$$

there exists $\Delta \mapsto \pmb \lambda (\Delta )$ on $(-1,1)$ such that

- for every $\Delta \in (-1,1)$, $\pmb \lambda (\Delta )$ is a solution to (19)
- $\Delta \mapsto \pmb \lambda (\Delta )$ is analytic on $(-1,1)$;
- for every $\Delta \in (-1,1)$ and $f\in L^1({\mathbb {R}})$,
  24 $$\begin{aligned} \Big |\frac{1}{N} \sum \limits _{j = 1}^n f\big ( \lambda _j (\Delta ) \big ) \, - \, \int \limits _{-{\mathfrak {q}} }^{ {\mathfrak {q}} } f(\lambda )\,\rho \big ( \lambda | {\mathfrak {q}} \big )d\lambda \Big | \le C_0\frac{\log N}{N}\,\Vert f' \Vert _{L^1({\mathbb {R}})}. \end{aligned}$$

In Remark 19, we will also see from the proof that one can obtain a solution $\pmb \lambda (-1)$ of (19) with $\Delta =-1$ by taking the limit of $\tfrac{1}{\zeta }\pmb \lambda (\Delta )$ when $\zeta$ tends to 0. This solution also satisfies (24).

**Organization** The paper is split into seven further sections and several appendixes. In Sects. 2 and 3, we present the proofs of Theorems 3 and 4, respectively. The sections themselves start with general considerations and are then divided into the different cases $0\le \Delta <1$, $-1< \Delta \le 0$ and $\Delta < -1$, as these exhibit different features. Sections 4 and 5 contain the proofs of Theorems 5 and 6, respectively.

Building upon these results, Theorems 1 and 2 are proved in Sects. 6 and 7, respectively. These sections are divided between the cases $|\Delta |<1$ and $\Delta < -1$ as these correspond to different behaviours.

Finally, Sect. 8 presents a refined version of Theorem 2. While being interesting in its own right, this result is mostly useful in our subsequent paper [17].

The first Appendix lists the different definitions of functions in order to have a place conveniently gathering all the formulae. The three other Appendixes gather properties of $\rho (\cdot |q)$ and (15) so as to not overburden the rest of the text.

## Proof of Theorem 3

In this whole section, fix $\Delta \in (-\infty ,-1)\cup (-1,1)$ and $n\le N/2$. Recall that *N* is even and that $I_i=i-\tfrac{n+1}{2}$ for $1\le i\le n$.

Below, we introduce the notion of an *interlaced solution* which will be useful in the proof. For $q > 0$ (with $q \le \pi /2$ when $\Delta <-1$) and $x\in (-x_0,x_0)$, where $x_0=x_0(q)\in {\mathbb {R}}_+\cup \{+\infty \}$ is defined by

$$\begin{aligned} \int \limits _0^\infty \rho (\lambda |q)d\lambda =\tfrac{1}{N}(x_0-\tfrac{n+1}{2}), \end{aligned}$$

introduce the quantile $\Lambda (x|q)$ given by the formula

25 $$\begin{aligned} \int \limits _0^{\Lambda (x|q)} \rho (\lambda |q)d\lambda =\tfrac{1}{N}(x-\tfrac{n+1}{2}). \end{aligned}$$

Note that $\Lambda (x|q)$ is unambiguously defined since $\rho (\lambda |q)>0$.

Due to the definition of $\rho (\cdot |q)$ (see “Appendix A” and (23)), $x_0$ is equal to infinity for $\Delta < -1$ and is finite, but larger than or equal to $\pi /2$ for $\Delta \ge -1$. In the latter case, in order to avoid unnecessarily heavy notation, we set $\Lambda (x|q)=+\infty$ for $x\ge x_0$ and $-\infty$ for $x\le -x_0$. Note also that by definition of ${\mathfrak {q}}$, *c.f.* (16) and (21), we have that ${\mathfrak {q}} = \Lambda ( n + \tfrac{1}{2} | {\mathfrak {q}}) = -\Lambda (\tfrac{1}{2}| {\mathfrak {q}})$.

### Definition 7

For $n\le \tfrac{N}{2}$, $k\ge 1$ and $q\in {\mathbb {R}}_+$, $\pmb \lambda =(\lambda _1,\dots , \lambda _n)\in {\mathbb {R}}^n$ is (*k*, *q*)-*interlaced* if for every $1\le i\le n$,

26 $$\begin{aligned} \Lambda (i-\tfrac{k}{2}|q)\le \lambda _i \le \Lambda (i+\tfrac{k}{2}|q). \end{aligned}$$

We say that $\pmb \lambda$ is (*k*, *q*)-*strictly interlaced* if the strict inequalities hold.

### Remark 8

When $k=1$, this corresponds to a perfect interlacement between the $\lambda _i$ and the quantiles of the measure $\rho (\lambda |q)d\lambda$.

The interest of this notion of interlacement becomes apparent in the following lemma which states that (*k*, *q*)-interlaced solutions satisfy (20), provided $k \le C/\zeta$.

### Lemma 9

(From interlacement to quantitative condensation). Fix $k\ge 1$ and $q\in {\mathbb {R}}_+$. For every (*k*, *q*)-interlaced $\pmb \lambda$ and every $f:{\mathbb {R}} \rightarrow {\mathbb {R}}$ with integrable derivative,

27 $$\begin{aligned} \Big |\frac{1}{N} \sum _{j = 1}^n f(\lambda _j) - \int \limits _{ \Lambda (\frac{1}{2}|q) }^{ \Lambda (n+\frac{1}{2}|q) }  f(\lambda ) \rho (\lambda | q)\, d\lambda \Big | \le \frac{k}{N} \Vert f' \Vert _{L^1( {\mathcal {I}}_k )}. \end{aligned}$$

with ${\mathcal {I}}_k\,{:=}\,( \Lambda (1-\tfrac{k}{2}|q) , \Lambda (n+\tfrac{k}{2}|q))$. Furthermore, if *f* is monotonic, the constant $k\Vert f' \Vert _{L^1({\mathcal {I}}_k)}$ can be replaced by

$$\begin{aligned} \tfrac{k+1}{2}\max \Big \{ \big | f(\lambda _1)-f\big ( \Lambda (n+\tfrac{1}{2}|q) \big ) \big |,\big |f(\lambda _n)-f\big ( \Lambda (\tfrac{1}{2}|q) \big ) \big | \Big \}. \end{aligned}$$

### Proof

By (25), the integral of $\rho (\cdot |q)$ between $\Lambda (j-\tfrac{1}{2}|q)$ and $\Lambda (j+\tfrac{1}{2}|q)$ is $\tfrac{1}{N}$, as long as both arguments are inbetween $-x_0$ and $x_0$. Thus, we find

$$\begin{aligned} \Big |\frac{1}{N} \sum _{j = 1}^n f(\lambda _j)- \int \limits _{ \Lambda (\frac{1}{2}|q) }^{ \Lambda (n+\frac{1}{2}|q) } f(\lambda )\rho (\lambda |q)d\lambda \Big |&\le \sum _{j = 1}^{n} \int \limits _{\Lambda (j-\frac{1}{2}|q)}^{\Lambda (j+\frac{1}{2}|q)}  |f(\lambda _j)-f(\lambda )|\rho (\lambda |q)d\lambda \\&\le \frac{1}{N}\sum _{j = 1}^n\int \limits _{\Lambda (j-\frac{k}{2}|q)}^{\Lambda (j+ \frac{k}{2}|q)}  |f'(\mu )|d\mu \le \frac{k}{N}\,\Vert f' \Vert _{L^1({\mathcal {I}}_k)}, \end{aligned}$$

where we invoked the following inequality, valid for $\Lambda (j-\frac{1}{2}|q)\le \lambda \le \Lambda (j+\frac{1}{2}|q)$,

$$\begin{aligned} |f(\lambda _j)-f(\lambda )|=\Big |\int \limits _\lambda ^{\lambda _j}f'(\mu )d\mu \Big |\le \int \limits _{\Lambda (j-\frac{k}{2}|q)}^{\Lambda (j+\frac{k}{2}|q)}|f'(\mu )|d\mu . \end{aligned}$$

In the case when *f* is non-decreasing (non-increasing works in the same way), (*k*, *q*)-interlacement gives

28 $$\begin{aligned} N  \int \limits _{\Lambda (j-\frac{k}{2}-1|q)}^{\Lambda (j-\frac{k}{2}|q)}  f(\lambda )\rho (\lambda |q)d\lambda \le f(\lambda _j)\le N \int \limits _{\Lambda (j+\frac{k}{2}|q)}^{\Lambda (j+\frac{k}{2}+1|q)}  f(\lambda )\rho (\lambda |q)d\lambda . \end{aligned}$$

The lower-bound holds for $n \ge j\ge (k+3)/2$ while the upper one for $1 \le j \le n-\tfrac{k+1}{2}$. Summing the left-hand side over $j\ge (k+3)/2$, bounding from below the remaining sums of $f(\lambda _j)$ by $\tfrac{k+1}{2}f(\lambda _1)$, and the missing piece of integral by $-\tfrac{k+1}{2}f\big ( \Lambda (n+\tfrac{1}{2}|q) \big )$ gives the lower bound on the difference. The upper bound follows from analogousconsiderations. $\square$

The core of the proof of Theorem 3 will be to construct solutions of (19) that lie in the subset of ${\mathbb {R}}^n$ given by

29 $$\begin{aligned} \Omega _{k,R}:=\{\text {strictly }(k, {\mathfrak {q}} )-\text { interlaced, symmetric, strictly ordered }\pmb \lambda \text { with }|\lambda _i|<R,\forall i\},\nonumber \\ \end{aligned}$$

with ${\mathfrak {q}}$ given by (21), as fixed points of a well-chosen function. This will be done by picking $k=k(\Delta )$ and $R=R(\Delta ,N)$ carefully, and then proving that the closure ${\overline{\Omega }}_{k,R}$ of $\Omega _{k,R}$ is mapped to $\Omega _{k,R}$ by this function. Then, the Brouwer fixed point theorem implies the existence of a fixed point for this function, which is a solution to (19) due to the choice of the function. While the proof is fairly similar in the different regimes, some tiny differences still exist and we therefore divide it between the cases $\Delta \in [0,1)$, $\Delta \in (-1,0]$, and $\Delta <-1$. Let us mention that a fixed point method was already used, although in a slightly different manner, for proving the existence of Bethe roots in [22].

### Remark 10

The reason for distinguishing between $\Delta \ge 0$ and $\Delta <0$ issues from the fact that $\lambda \mapsto \vartheta (\lambda )$ given in “Appendix A” is respectively decreasing and increasing. Furthermore, when $\Delta <0$, dividing between $\Delta <-1$ and $\Delta >-1$ comes from the small caveat that the image of $\vartheta$ is an interval of length strictly smaller than $2\pi$ when $\Delta >-1$ and equal to $2\pi$ when $\Delta < -1$.

### Remark 11

We expect the existence of $(1,{\mathfrak {q}})$-interlaced solutions to (19) for every $\Delta <1$, even though we are currently unable to prove this fact for *n* close to *N*/2 (when $n/N\le 1/2-\epsilon$, this follows readily from the results established in [28]). Below are plots of

$$\begin{aligned} {\mathbf {m}}:=\max \Big \{ N\int _{\Lambda (i| {\mathfrak {q}})}^{\lambda _i} \rho (\lambda |\infty ) d\lambda \; : \; 1\le i\le N/2 \Big \} \end{aligned}$$

as a function of the system size *N*, for $n=N/2$ and different $\Delta \ge -1$. One sees that the quantity remains bounded by 1/2, meaning that the solution is $(1,+\infty )$-interlaced.
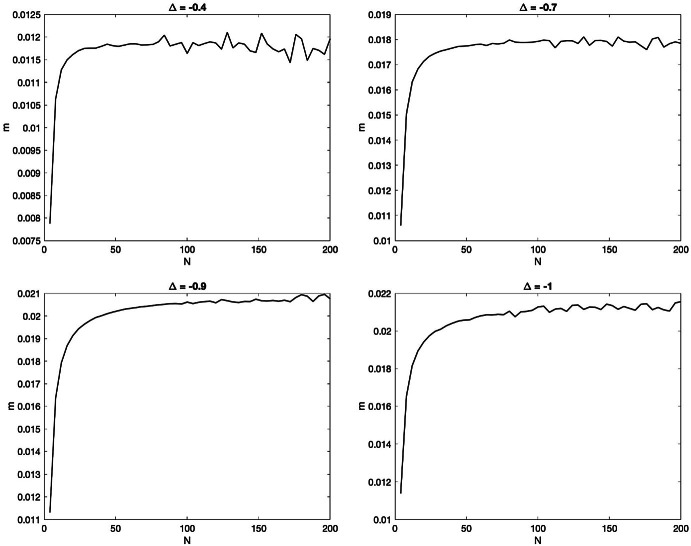


### Case $0\le \Delta < 1$

Introduce the smallest positive integer $k=k(\Delta )$ such that

30 $$\begin{aligned} 2\pi \frac{k}{k+1}>|\vartheta (+\infty )-\vartheta (-\infty )|=|2\pi -4\zeta |. \end{aligned}$$

Consider the map $\Phi :{\mathbb {R}}^n\rightarrow {\mathbb {R}}^n,\pmb \lambda \mapsto \pmb \mu$ for which $\mu _i$ is defined for every $1\le i\le n$ by

31 $$\begin{aligned} {\mathfrak {p}}(\mu _i)-\tfrac{1}{N}\sum _{j=1}^n\vartheta (\mu _i-\lambda _j)=\tfrac{2\pi }{N} I_i, \end{aligned}$$

where ${\mathfrak {p}}$ and $\vartheta$ are given in “Appendix A”.

This function is well-defined as the map $x\mapsto {\mathfrak {p}}(x)-\tfrac{1}{N}\sum _{j=1}^n\vartheta (x-\lambda _j)$ is continuous strictly increasing (here the fact that $\Delta \ge 0$ ensures that $\vartheta$ is decreasing) and tends to $\pm \Upsilon$ with $\Upsilon := (1-\tfrac{n}{N})\pi - \zeta (1-2\tfrac{n}{N})$ at $\pm \infty$. Also, since $|\tfrac{2\pi }{N} I_i|\le \tfrac{\pi }{2}-\tfrac{\pi }{N}$ and $\Upsilon \ge \tfrac{\pi }{2}$ (recall that $n \le N/2$ and that $\zeta \le \pi /2$ in this case), there exists a constant $R=R(\Delta ,N)$ such that $|\mu _i|< R$ for every *i*. From now on, we fix this constant and show that $\Phi$ maps ${\overline{\Omega }}_{k,R}$ onto $\Omega _{k,R}$. The Brouwer fixed point theorem then implies that $\Phi$ has a fixed point (since ${\overline{\Omega }}_{k,R}$ is a compact convex set), which is a strictly $(k, {\mathfrak {q}})$-interlaced symmetric strictly ordered solution of (19). Note that the choice (30) of *k* ensures that $k \le C/\zeta$ for some universal constant *C*, which implies (20) through Lemma 9.

Let $\pmb \mu = \Phi (\pmb \lambda )$ for some $\pmb \lambda \in {\overline{\Omega }}_{k,R}$. That $\pmb \mu$ is strictly ordered and symmetric is obvious from (31), whose left-hand side is strictly increasing in $\mu _i$. We therefore only need to check the strict $(k,{\mathfrak {q}})$-interlacement, which is a direct consequence of the following sequence of inequalities:

32 $$\begin{aligned} 2\pi \Big |N\int \limits _0^{\mu _i}\rho (\lambda |{\mathfrak {q}})d\lambda -I_i\Big |&=\Big |N{\mathfrak {p}}(\mu _i)-N\int \limits _{-{\mathfrak {q}}}^{{\mathfrak {q}}}\vartheta (\mu _i-\lambda )\rho (\lambda |{\mathfrak {q}})d\lambda -2\pi I_i\Big |\nonumber \\&\le \tfrac{k+1}{2}|\vartheta (+\infty )-\vartheta (-\infty )|{\mathop {<}\limits ^{(30)}}\pi k. \end{aligned}$$

The equality in (32) is due to the following identity, valid for every *x* and *q*,

33 $$\begin{aligned} 2\pi \int \limits _0^{x} \rho (t|q)dt={\mathfrak {p}}(x)-\int \limits _{-q}^q\vartheta (x-\lambda )\rho (\lambda |q)d\lambda , \end{aligned}$$

which is the integrated version of (15) (recall that $\vartheta$ and ${\mathfrak {p}}$ are odd). The first inequality in (32) is an application of the definition (31) of $\mu _i$ together with Lemma 9 applied to the monotone function $\vartheta (\mu _i-\cdot )$; it is also useful to recall that ${\mathfrak {q}}=\Lambda ( n+\tfrac{1}{2} |{\mathfrak {q}}) = - \Lambda ( 1 - \tfrac{1}{2} |{\mathfrak {q}})$, due to the definitions of ${\mathfrak {q}}$ and $\Lambda (\cdot |q)$.

### Case $-1<\Delta <0$

As before, introduce the smallest positive integer $k=k(\Delta )$ such that (30) holds.

We are unable to use the map $\Phi$ from the previous subsection as $\vartheta$ is now increasing. We therefore change the map slightly and consider the map $\Psi :{\mathbb {R}}^n\rightarrow {\mathbb {R}}^n,\pmb \lambda \mapsto \pmb \mu$ for which $\mu _i$ is defined for every $1\le i\le n$ by

34 $$\begin{aligned} {\mathfrak {p}}(\mu _i) = \tfrac{1}{N} \sum _{j=1}^n\vartheta (\lambda _i-\lambda _j)+\tfrac{2\pi }{N} I_i. \end{aligned}$$

The map is again well-defined as ${\mathfrak {p}}$ is continuous, strictly increasing, and ${\mathfrak {p}}({\mathbb {R}})$ is equal to $[\zeta -\pi , \pi - \zeta ]$, while for any $\pmb \lambda \in {\mathbb {R}}^n$,

$$\begin{aligned} - \tfrac{2n}{N} (\pi -\zeta ) + \tfrac{\pi }{N} \,< \, \tfrac{1}{N}\sum _{j=1}^n\vartheta (\lambda _i-\lambda _j)+\tfrac{2\pi }{N} I_i \, < \, \tfrac{2n}{N} (\pi -\zeta ) - \tfrac{\pi }{N} \;, \end{aligned}$$

(we use that $|\vartheta |\le \pi -2\zeta$, $|I_i|\le (n-1)/2$) which ensures that the left-hand side of (34) lies in the range of ${\mathfrak {p}}$ since $n\le N/2$. Thus, as before, we may find $R=R(\Delta ,N)$ large enough such that $|\mu _i|< R$ for every *i*.

Again, we wish to prove that $\Psi$ is mapping ${\overline{\Omega }}_{k,R}$ to $\Omega _{k,R}$, which will imply the existence of a fixed point, and therefore a strictly $(k,{\mathfrak {q}})$-interlaced symmetric, strictly ordered solution to (19). Note that definition (30) for *k* entails that $k \le C/\zeta$ for some constant $C>0$, which implies (20) by applying Lemma 9.

Fix $\pmb \lambda \in {\overline{\Omega }}_{k,R}$ and set $\pmb \mu := \Psi (\pmb \lambda )$. The strict monotonicity and the fact that $\mu _i\in (-R,R)$ are immediate consequences of the definition of $\Psi$ and the choice of *R*, and we do not give further details. Lemma 9 applied to the decreasing function $\vartheta (\lambda _i-\cdot )$ implies that

$$\begin{aligned} N{\mathfrak {p}}(\mu _i)&\le N\int \limits _{-{\mathfrak {q}}}^{{\mathfrak {q}}}\vartheta (\lambda _i-\lambda )\rho (\lambda |{\mathfrak {q}})d\lambda \\&\quad + \tfrac{k+1}{2} \max \big \{ | \vartheta (\lambda _i-\lambda _1)-\vartheta (\lambda _i-{\mathfrak {q}})|, | \vartheta (\lambda _i-\lambda _n)-\vartheta (\lambda _i+{\mathfrak {q}})| \big \} +2\pi I_i. \end{aligned}$$

Observe now that, due to (30), the maximum above is smaller than $|2\pi -4\zeta | < 2\pi \frac{k}{k+1}$. Since in addition $\lambda _i$ was assumed smaller than $\Lambda (i+\tfrac{k}{2}|{\mathfrak {q}})$, and since $\vartheta$ is increasing, we conclude that

$$\begin{aligned} N{\mathfrak {p}}(\mu _i)&< N\int \limits _{-{\mathfrak {q}}}^{{\mathfrak {q}}}\vartheta ( \Lambda (i+\tfrac{k}{2}|{\mathfrak {q}} )-\lambda )\rho (\lambda |{\mathfrak {q}})d\lambda +2\pi I_{i} +2\pi \tfrac{k}{2}=N{\mathfrak {p}}\big ( \Lambda (i+\tfrac{k}{2}|{\mathfrak {q}}) \big ), \end{aligned}$$

where the last equality follows from (33) and the definition of $\Lambda (i+\tfrac{k}{2}|{\mathfrak {q}})$. Since ${\mathfrak {p}}$ is increasing, we get that $\mu _i< \Lambda (i+\tfrac{k}{2}|{\mathfrak {q}})$. Similarly, one proves that $\mu _i> \Lambda (i-\tfrac{k}{2}|{\mathfrak {q}})$.

### Case $\Delta < -1$

For $\Delta < -1$ and $q>0$, first observe that the function $\varphi (\lambda ):=2 \vartheta (\lambda )-\vartheta (\lambda +\tfrac{\pi }{2})-\vartheta (\lambda -\tfrac{\pi }{2})$ is increasing on $[0,\pi /4]$ and decreasing on $[\pi /4,\pi /2]$ with $\varphi (0)=\varphi (\pi /2)=0$ and $\varphi (\pi /4)\in (0,2\pi )$. Moreover, $\varphi$ is $\pi$-periodic and even (due to the corresponding properties for $\vartheta$), and therefore $\varphi (\pi /4)$ is its maximum over all ${\mathbb {R}}$. Then, introduce the smallest *odd* integer $k\in {\mathbb {Z}}_+$ such that

35 $$\begin{aligned} 2\pi \frac{k}{k+1}>\sup \big \{ |\varphi (\lambda )| \, : \, \lambda \in {\mathbb {R}} \big \} = \varphi ( \tfrac{\pi }{4}) \,. \end{aligned}$$

In the present case, we reuse the map $\Psi$ defined in Sect. 2.2. This map is well defined since, in this regime of $\Delta$, ${\mathfrak {p}}$ is strictly increasing and ${\mathfrak {p}}({\mathbb {R}})={\mathbb {R}}$.

For small values of *N*, the existence of a fixed point of $\Psi$ (or equivalently of a solution to (19)) that is not necessarily (*k*, *R*)-interlaced is easily obtained. Its condensation may be derived by adjusting the constant *C* in (20). Henceforth we focus on values of *N* above a threshold independent of $\Delta$ chosen below.

Let $\pmb \mu = \Psi (\pmb \lambda )$ for some $\pmb \lambda \in {\overline{\Omega }}_{k,R}$. As in the previous part, it is immediate that $\pmb \mu$ is symmetric and strictly ordered. One should still establish the boundedness and the strict $(k,{\mathfrak {q}})$-interlacement of $\pmb {\mu }$. We will argue that the former is a direct consequence of the later. We thus first establish interlacement.

To do so, one should start by establishing a generalisation of Lemma 9 to the case of a function $g :[0,+\infty ) \rightarrow {\mathbb {R}}$ which is monotonous on $[0,\pi /2]$. Here, we only treat the case of *n* even and leave the details of *n* odd to the reader, since it only leads to minor modifications. We claim that, for any such function *g*,

36 $$\begin{aligned} \Big | \sum _{i=1+ \frac{n}{2} }^{ n } g(\lambda _i) \, - \, N \int \limits _{0}^{{\mathfrak {q}}} g(\mu ) \rho (\mu |{\mathfrak {q}}) d \mu \Big | \, \le \, \frac{k+1}{2} \text {max}\big \{ {\mathfrak {m}}^+[g], {\mathfrak {m}}^-[g] \big \}, \end{aligned}$$

where

$$\begin{aligned} {\mathfrak {m}}^+[g] := \text {max} \big \{ | g(\lambda _j)-g(0) | \, : \, j=n-\tfrac{k-1}{2},\dots , n \big \} \;\; \text {and} \;\; {\mathfrak {m}}^-[g] := | g( {\mathfrak {q}} )- g( \lambda _{\frac{n}{2}+1} )| . \end{aligned}$$

The inequality above is obtained in the same way as Lemma 9, so we provide no further details.

Define $\vartheta ^\mathrm{sym}(\lambda ,\mu ):=\vartheta (\lambda -\mu )+\vartheta (\lambda +\mu )$. Then a direct computation shows that the functions $\vartheta ^\mathrm{sym}(\lambda _i,\cdot )$ for $i = 1,\dots , n$ are monotonous on $[0,\pi ]$. Applying (36) to $\vartheta ^\mathrm{sym}(\lambda _i,\cdot )$ we find

37 $$\begin{aligned} {\mathfrak {p}}(\mu _i)&= \tfrac{1}{N} \sum _{j=1+ \frac{n}{2} }^{n}\vartheta ^\mathrm{sym}(\lambda _i,\lambda _j)+\tfrac{2\pi }{N} I_i \nonumber \\&\ge \int \limits _{0}^{{\mathfrak {q}}} \vartheta ^\mathrm{sym}(\lambda _i,\mu )\rho (\mu |{\mathfrak {q}}) d \mu \,-\,\tfrac{k+1}{2N} \nonumber \\&\quad \times \text {max}\big \{{\mathfrak {m}}^+[\vartheta ^\mathrm{sym}(\lambda _i,\cdot )], {\mathfrak {m}}^-[\vartheta ^\mathrm{sym}(\lambda _i,\cdot )]\big \}+\tfrac{2\pi }{N} I_i . \end{aligned}$$

It follows from the lower bound $\rho (x|q)\ge \rho (x)\ge \frac{1}{2\zeta }$ established in Lemma 29 of “Appendix D”, that for each *j*

38 $$\begin{aligned} \Lambda (n+\tfrac{1}{2}|{\mathfrak {q}}) \, - \, \Lambda (n-j +\tfrac{1}{2} | {\mathfrak {q}}) \le \;2\zeta  \int \limits _{\Lambda (n-j+\frac{1}{2}|{\mathfrak {q}})}^{\Lambda (n+\frac{1}{2}|{\mathfrak {q}})}\rho (\lambda |{\mathfrak {q}})d\lambda = 2 j\tfrac{\zeta }{N}. \end{aligned}$$

Therefore, the $(k,{\mathfrak {q}})$-interlacement of $\pmb \lambda$ allows one to infer that $\lambda _j={\mathfrak {q}} + O(\tfrac{k}{N})$, with the *O*(.) here and below being uniform in $j=n-\tfrac{k-1}{2},\dots , n$ and $\Delta < -1$. Hence, since ${\mathfrak {q}}\le \pi /2$, any $\lambda _j$ appearing in the definition of ${\mathfrak {m}}^+[g]$ exceeds $\pi /2$ by at most $O(\tfrac{k}{N})$.

A direct computation shows that $\pi /2$ is a local extremum of $\mu \mapsto \vartheta ^\mathrm{sym}(\lambda ,\mu )$ on $[\pi /2-\eta , \pi /2+\eta ]$ for some $\eta >0$ independent of $\Delta$ and $\lambda$. Thus, we conclude that for all *N* large enough (which we will assume henceforth for reasons described at the start of the proof), every $2n\le N$ and $i\in \{1+\tfrac{n}{2},\dots , n\}$,

$$\begin{aligned} \text {max}\big \{{\mathfrak {m}}^+[ \vartheta ^\mathrm{sym}(\lambda _i,\cdot ) ], {\mathfrak {m}}^-[ \vartheta ^\mathrm{sym}(\lambda _i,\cdot ) ]\big \} \le | \vartheta ^\mathrm{sym}(\lambda _i, \tfrac{\pi }{2} ) - \vartheta ^\mathrm{sym}(\lambda _i, 0 ) | = |\varphi (\lambda _i) | . \end{aligned}$$

Plugging the above into (37), we find

$$\begin{aligned} {\mathfrak {p}}(\mu _i) \ge \int \limits _{0}^{{\mathfrak {q}}} \vartheta ^\mathrm{sym}(\lambda _i,\mu )\rho (\mu |{\mathfrak {q}}) d \mu \, - \, \tfrac{k+1}{2N}\varphi (\tfrac{\pi }{4}) +\tfrac{2\pi }{N} I_i . \end{aligned}$$

Invoking the choice of *k* and the fact that $\lambda \mapsto \vartheta (\lambda )$ is increasing on ${\mathbb {R}}$ gives that

$$\begin{aligned} {\mathfrak {p}}(\mu _i) > \int \limits _{ - {\mathfrak {q}} }^{ {\mathfrak {q}} } \vartheta \big ( \Lambda (n-\tfrac{k}{2}| {\mathfrak {q}}) - \mu \big ) \rho (\mu |{\mathfrak {q}}) d \mu \, + \, \tfrac{2\pi }{N} I_{i-\frac{k}{2}} \, = \, {\mathfrak {p}}( \Lambda (n-\tfrac{k}{2}| {\mathfrak {q}}) ) . \end{aligned}$$

This yields the lower bound for the $(k,{\mathfrak {q}})$-interlacement of $\pmb {\mu }$. The upper bound is obtained in an analogous way.

Finally, the $(k,{\mathfrak {q}})$-interlacement of $\pmb {\mu }$ and the upper bound $\Lambda (n+\tfrac{k}{2}| {\mathfrak {q}})\, - \, \Lambda (n+\tfrac{1}{2}|{\mathfrak {q}}) \le \frac{\zeta (k-1)}{N}$ ensure that $\mu _n\le {\mathfrak {q}} + \tfrac{\zeta (k-1)}{N}$ and thus, by symmetry, that $\mu _i \in (-R,R)$ with $R:=\tfrac{\pi }{2}+\frac{\zeta (k-1)}{N}$. The latter establishes that $\Psi ( \overline{\Omega }_{k,R})\subset \Omega _{k,R}$.

As before, we deduce through Brouwer’s fixed point theorem that $\Psi$ admits a fixed point, which provides a solution to (19) satisfying the conditions of Theorem 3. The validity of (20) is due to Lemma 9 and the fact that $k \le C/\zeta$ for some universal constant *C*; the latter follows directly from (35) and an upper bound on $\varphi (\tfrac{\pi }{4})$ easily obtained from the definition of $\vartheta$ and “Appendix A.3”.

## Proof of Theorem 4

Fix $n\le N/2$. Since the dependence on $\Delta$ plays a role in this argument, we recall it in the subscript of the map ${\mathsf {T}}:(\Delta ,\pmb \lambda )\mapsto {\mathsf {T}}_\Delta (\pmb \lambda )$ from $[-\infty , 1) \times {\mathbb {R}}^n$ to ${\mathbb {R}}^n$ defined by the formula

39 $$\begin{aligned} \big [ {\mathsf {T}}_\Delta (\pmb \lambda )\big ]_i =\tfrac{1}{2\pi }{\mathfrak {p}} (\lambda _i) - \tfrac{1}{2\pi N} \sum _{j = 1}^{n} \vartheta (\lambda _i-\lambda _j)-\tfrac{1}{N}I_i \; , \quad 1 \le i \le n . \end{aligned}$$

We recall that ${\mathfrak {p}}$ and $\vartheta$ appearing above do depend on $\Delta$, *c.f.* “Appendix A”.

The zeroes of ${\mathsf {T}}_\Delta$ correspond to the solutions to (19) for $\Delta$. The following proposition will play a key role in the proof of Theorem 4.

### Proposition 12

Let *k* be as defined by (30) for $-1< \Delta < 1$ and (35) for $\Delta <-1$. Then, there exists some universal constant *C* such that, for every $\Delta \in (-\infty , 1){\setminus } \{-1\}$, every *N* large enough, and every

$$\begin{aligned} n\le N/2-Ck^2 \pmb { \mathbb {1} }_{(-1,0)}(\Delta ) \, , \end{aligned}$$

we have that $d{\mathsf {T}}_\Delta$ is invertible at $\pmb \lambda$ for any $(k, {\mathfrak {q}})$-interlaced, ordered, symmetric $\pmb \lambda$.

With this proposition at hand, we are in position to prove the theorem.

### Proof of Theorem 4

Taking into account the definition of ${\mathsf {T}}_\Delta$ and introducing $\Omega (\Delta ):=\Omega _{k(\Delta ),R(\Delta ,N)}$, with $\Omega _{k,R}$ given by (29) and $R(\Delta ,N)$ as constructed in Sects. 2.1, 2.2 and 2.3, depending on the value of $\Delta$, we can restate the theorem as the existence of an analytic family $\Delta \mapsto \pmb \lambda (\Delta )$ such that $\pmb \lambda (\Delta )\in \Omega (\Delta )$ satisfies ${\mathsf {T}}_\Delta (\pmb \lambda (\Delta ))=0$ for every $\Delta$.

Consider some $\Delta _0$ for which we are in the possession of $\pmb \lambda (\Delta _0)\in \Omega (\Delta _0)$ satisfying ${\mathsf {T}}_{\Delta _0}(\pmb \lambda (\Delta _0))=0$. Using Proposition 12, the implicit function theorem for analytic functions gives the existence of an analytic family $\Delta \mapsto \pmb \lambda (\Delta )\in {\mathbb {R}}^n$ such that ${\mathsf {T}}_\Delta (\pmb \lambda (\Delta ))=0$ in a small neighbourhood of $\Delta _0$. Continuity implies that by reducing the neighbourhood if need be, we can further assume that $\pmb \lambda (\Delta )\in \Omega (\Delta )$.

Also note that a continuous limit, as $\Delta$ tends to some $\Delta _1$, of $\pmb \lambda (\Delta )\in \Omega (\Delta )$ with ${\mathsf {T}}_\Delta (\pmb \lambda (\Delta ))=0$ converges to $\pmb \lambda (\Delta _1)\in {\overline{\Omega }}(\Delta _1)$ with ${\mathsf {T}}_{\Delta _1}(\pmb \lambda (\Delta _1))=0$. But, we saw in the previous section that solutions to (19) in ${\overline{\Omega }}(\Delta _1)$ are necessarily in $\Omega (\Delta _1)$. Together with the previous paragraph, this implies the existence of an analytic family of solutions on any open interval on which the conditions of Proposition 12 hold, and which contains at least one value $\Delta$ for which there exists a solution $\pmb \lambda \in \Omega (\Delta )$ to (19). The intervals of Theorem 4 are indeed such that the conditions of Proposition 12 hold; the existence of solutions for some $\Delta$ in these intervals is ensured by Theorem 3 (or alternatively by Lemmata 16 and 17, see below).

To prove the uniqueness of the solutions for all $\Delta$, it suffices to prove it for a single value $\Delta _1$ in each of the two intervals of Theorem 4. Indeed, assuming the existence of multiple solutions at some value of $\Delta$, the argument above implies the existence of multiple analytic families of solutions in the whole interval. These families may not cross inside the interval, due to the implicit function theorem, and would therefore contradict the uniqueness at $\Delta _1$. We choose to check the uniqueness of solutions for $\Delta _1=0$ and $\Delta _1$ a very large negative number. This is done by solving (19) explicitly for $\Delta =0$ and $\Delta =-\infty$, then extending the property to large negative numbers by continuity; see Lemmata 16 and 17 below for more details. $\square$

We now focus on the proof of Proposition 12, and divide it into three subsections depending on the range of $\Delta$ as before. Note that since $K=\tfrac{1}{2\pi }\vartheta '$ and $\xi =\tfrac{1}{2\pi }{\mathfrak {p}}'$, the matrix $d{\mathsf {T}}_{\Delta } (\pmb \lambda )$ can be evaluated as

$$\begin{aligned} \Big [ d{\mathsf {T}}_{\Delta }(\pmb \lambda ) \Big ]_{ij}= {\left\{ \begin{array}{ll} \displaystyle \xi (\lambda _i) -\frac{1}{N} \sum _{\ell \not = i}K(\lambda _i - \lambda _\ell ) \qquad &{} i=j,\\ \displaystyle \frac{K(\lambda _i - \lambda _j)}{N} \qquad &{} i\ne j. \end{array}\right. } \end{aligned}$$

### Proof of Proposition 12 when $0\le \Delta <1$

For any $\pmb \lambda \in {\mathbb {R}}^n$, the matrix $d{\mathsf {T}}_{\Delta }(\pmb \lambda )$ is symmetric (since *K* is even) and positive definite:

$$\begin{aligned} \big ( {\varvec{v}} , d{\mathsf {T}}_{\Delta }(\pmb \lambda ) {\varvec{v}} \big ) \,&= \, \sum \limits _{i=1}^{N}\Big ( \xi (\lambda _i) -\frac{1}{N} \sum _{\ell \not = i}K(\lambda _i - \lambda _\ell ) \Big ) v_i^2 \, + \, \sum \limits _{i\not =j }^{ N} v_i v_j \frac{K(\lambda _i - \lambda _j)}{N} \\ \;&= \; \sum \limits _{i=1}^{N} \xi (\lambda _i) v_i^2 - \frac{1}{2N} \sum \limits _{i\not =j }^{ N} (v_i -v_j)^2 K(\lambda _i - \lambda _j) \; \ge \; 0, \end{aligned}$$

since $K\le 0$ and $\xi > 0$, as these are derivatives of decreasing and strictly increasing functions, respectively (see also “Appendix A”). As a consequence, $d{\mathsf {T}}_{\Delta }(\pmb \lambda )$ is invertible.

#### Remark 13

Alternatively, in this regime, one can obtain existence and uniqueness of the solution to the Bethe equation (19) as follows. Since $d{\mathsf {T}}_{\Delta }(\pmb \lambda )$ is a positive definite matrix, the Yang-Yang action [41]

$$\begin{aligned} S(\pmb \lambda ):=\sum _{i=1}^n \Big (\int \limits _0^{\lambda _i}{\mathfrak {p}}(\mu )d\mu -\frac{1}{2N}\sum _{j=1}^n\int \limits _0^{\lambda _i-\lambda _j}\vartheta (\mu )d\mu -\frac{2\pi I_i}{N}\lambda _i\Big ) \end{aligned}$$

is strictly convex, and has therefore at most one extremum which, if it exists, is its minimum. The existence thereof can be obtained in at least three ways. Either one uses the fixed point theorem in the previous section, or one checks that *S* tends to infinity as soon as one of the $\lambda _i$ tends to infinity (this is slightly technical), or finally one observes that at $\Delta =0$ there is an explicit solution and that the implicit function theorem guarantees that this solution extends into an analytic function on $0\le \Delta < 1$.

### Proof of Proposition 12 when $-1<\Delta <0$

We remind to the reader that in this regime we restrict our attention to *n* satisfying

40 $$\begin{aligned} n\le N/2-Ck^2, \end{aligned}$$

where *k* is given by (30) while *C* (which is independent of *N* and *k*) is yet to be determined. Also, we recall that ${\mathfrak {q}}$ is given by (21).

Fix $\pmb \lambda$ as in the proposition. The matrix $d{\mathsf {T}}_{\Delta }(\pmb \lambda )$ is no longer obviously positive definite and therefore not obviously invertible. In order to prove invertibility, we rather show that the matrix *A* defined by

$$\begin{aligned} A_{ij}:=\frac{d{\mathsf {T}}_{\Delta }(\pmb \lambda )_{ij}}{\rho (\lambda _j|{\mathfrak {q}})}= {\left\{ \begin{array}{ll} \displaystyle \frac{1}{\rho (\lambda _i|{\mathfrak {q}})} \big [\xi (\lambda _i) -\frac{1}{N} \sum _{ \begin{array}{c} \ell =1 \\ \not = i \end{array} }^{n}K(\lambda _i - \lambda _\ell )\big ]\qquad &{} i=j,\\ \displaystyle \frac{K(\lambda _i - \lambda _j)}{N\rho (\lambda _j|{\mathfrak {q}})} \qquad &{} i\ne j, \end{array}\right. } \end{aligned}$$

is diagonal dominant^5^ hence invertible (note that Proposition 24(i) of “Appendix B” gives that $\rho (\lambda |{\mathfrak {q}})>0$ on ${\mathbb {R}}$ and therefore the matrix *A* is well-defined). The invertibility of $d{\mathsf {T}}_{\Delta }(\pmb \lambda )$ follows trivially from that of *A*. Checking diagonal dominance relies on two computations.

On the one hand, Lemma 9 applied to $\lambda \mapsto K(\lambda _j-\lambda )$ (since $\pmb \lambda$ is $(k,{\mathfrak {q}})$-interlaced) together with (15) gives

41 $$\begin{aligned} A_{jj}&\ge \frac{1}{\rho (\lambda _j|{\mathfrak {q}})} \big (\xi (\lambda _j) - \int _{-{\mathfrak {q}}}^{\mathfrak {q}} K(\lambda _j-\lambda )\rho (\lambda | {\mathfrak {q}}) d\lambda - \frac{k}{N}\Vert K'\Vert _{L^1({\mathbb {R}})} \big ) \nonumber \\&= 1-\frac{k\Vert K'\Vert _{L^1({\mathbb {R}})}}{N\rho (\lambda _j|{\mathfrak {q}})}. \end{aligned}$$

On the other hand, Lemma 9 applied to the function

$$\begin{aligned} \lambda \mapsto f_j(\lambda ) :=K(\lambda _j-\lambda )/\rho (\lambda |{\mathfrak {q}}) \end{aligned}$$

gives^6^

42 $$\begin{aligned} \sum _{\ell \not = j} A_{j \ell }&= \frac{1}{N}\sum _{\ell \not = j} \frac{K(\lambda _j - \lambda _\ell )}{ \rho (\lambda _\ell | {\mathfrak {q}})} \nonumber \\&\le \int _{-{\mathfrak {q}}}^{\mathfrak {q}} \frac{K(\lambda _j - \lambda )}{\rho (\lambda | {\mathfrak {q}})} \rho (\lambda | {\mathfrak {q}}) d\lambda + \frac{k}{N}\Vert f_j'\Vert _{L^1({\mathcal {I}}_k)}\nonumber \\&= \frac{ \vartheta (\lambda _j+{\mathfrak {q}}) - \vartheta (\lambda _j-{\mathfrak {q}})}{2\pi } + \frac{k}{N} \Vert f_j'\Vert _{L^1({\mathcal {I}}_k)}\nonumber \\&{\mathop {\le }\limits ^{(30)}}\frac{k}{k+1}+ \frac{k}{N}\Vert f_j'\Vert _{L^1({\mathcal {I}}_k)}, \end{aligned}$$

where $${\mathcal {I}}_k:=[ \Lambda (1-\tfrac{k}{2}|{\mathfrak {q}}) , \Lambda (n+\tfrac{k}{2}|{\mathfrak {q}}) ]$$.

Now, we estimate the error terms (meaning the terms with factor $\tfrac{k}{N}$) in (41) and (42) separately. Below, the constants $C_i$ are independent of everything else. We use analytic properties of the solution to the continuum Bethe Equation (15) that are proved in Proposition 24 of “Appendix B”. The assumption (40) plays an essential role in what follows.

We start by estimating the error in (41). Using the fact that $|\rho ^{\prime }(\lambda )| \le \pi \rho (\lambda )/\zeta$ (see “Appendix A”) and further invoking Proposition 24(i), we find:

43 $$\begin{aligned} \rho (-{\mathfrak {q}})-\rho (\Lambda (1-\tfrac{k}{2}|{\mathfrak {q}}))&\le \tfrac{ \pi }{\zeta }\int _{\Lambda (1-k/2|{\mathfrak {q}})}^{\Lambda (1/2|{\mathfrak {q}})} \rho (\lambda )d\lambda \nonumber \\&\le \tfrac{\pi }{\zeta }\int _{\Lambda (1-k/2|{\mathfrak {q}})}^{\Lambda (1/2|{\mathfrak {q}})} \rho (\lambda |{\mathfrak {q}})d\lambda = \tfrac{\pi }{\zeta }\tfrac{k-1}{2N}. \end{aligned}$$

Furthermore, by Proposition 24(iii)

44 $$\begin{aligned} \rho (-{\mathfrak {q}}) \ge \tfrac{c_1}{\zeta }( \tfrac{1}{2} - \tfrac{n}{N} ) \quad \text {with} \quad c_1>0. \end{aligned}$$

Combining the two last displayed equations, we infer a lower bound

45 $$\begin{aligned} \rho (\Lambda (1-\tfrac{k}{2}|{\mathfrak {q}}))\ge \tfrac{c_1}{\zeta }(\tfrac{1}{2}-\tfrac{n+C_1k}{N})\quad \mathrm {with} \quad C_1: = \frac{\pi }{2c_1}. \end{aligned}$$

Then Proposition 24(i), the monotonicity of $\rho (\cdot )$ on $(-\infty , 0]$ and the symmetry and $(k,{\mathfrak {q}})$-interlacement of $\pmb \lambda$ give that

46 $$\begin{aligned} \rho (\lambda _j|{\mathfrak {q}})\ge \rho (\lambda _j)\ge \rho (\lambda _1)\ge \rho (\Lambda (1-\tfrac{k}{2}|{\mathfrak {q}}))\ge \tfrac{c_1}{\zeta }(\tfrac{1}{2}-\tfrac{ n + C_1 k}{N}) . \end{aligned}$$

Now, since *K* is unimodal, even, and has limits 0 at $\pm \infty$, $\Vert K'\Vert _{L^1({\mathbb {R}})}= 2K(0)$. Using this and the previous paragraph, we find

47 $$\begin{aligned} \frac{k}{N}\frac{\Vert K'\Vert _{L^1({\mathbb {R}})}}{\rho (\lambda _j|{\mathfrak {q}})} \le \frac{ 2\zeta K(0) k }{ c_1 (N/2-n-C_1k ) } \le \frac{ C_2 k }{ N/2-n-C_1k} \;, \end{aligned}$$

where the last inequality is obtained by observing that $K(0)\le C_0/\zeta$ for some $\zeta$-independent constant $C_0$.

We now turn to the error term in (42). First, we have that for $1\le j\le n$, $t\in {\mathcal {I}}_k$, and *N* large enough,

48 $$\begin{aligned} |f_j'(t)|&\le \frac{|K'(\lambda _j-t)|}{\rho (t|{\mathfrak {q}})}+|K(\lambda _j-t)|\frac{|\rho '(t|{\mathfrak {q}})|}{\rho (t|{\mathfrak {q}})^2}\nonumber \\&\le \frac{ |K'(\lambda _j-t)| + |K(\lambda _j-t)| \frac{C_3}{\zeta } (1+\frac{\rho ({\mathfrak {q}})}{\rho (t|{\mathfrak {q}})}) }{\rho (t|{\mathfrak {q}})}, \end{aligned}$$

where in the second inequality we used Proposition 24(ii).

Now, note that by Proposition 24(i) and (45), for every $t\in {\mathcal {I}}_k$,

49 $$\begin{aligned} \frac{1}{\rho (t|{\mathfrak {q}})} \le \frac{1}{\rho (t)} \le \frac{1}{\rho (\Lambda (1-\tfrac{k}{2}|{\mathfrak {q}}))} \le \frac{\tfrac{1}{c_1}\zeta N}{N/2 -n-C_1 k}. \end{aligned}$$

Also, Proposition 24(iii) gives that

50 $$\begin{aligned} \rho ({\mathfrak {q}}) \le \tfrac{C_4}{\zeta }( \tfrac{1}{2} - \tfrac{n}{N} ) \quad \text {with} \quad C_4>0. \end{aligned}$$

Plugging (49) and (50) in (48) and then integrating over ${\mathcal {I}}_k$, we find that

$$\begin{aligned} \frac{k}{N}\Vert f_j'\Vert _{L^1({\mathcal {I}}_k)}&\le \frac{k}{N}\frac{\tfrac{1}{c_1}\zeta N}{N/2 -n-C_1 k}\Big \{ \Vert K^{\prime }\Vert _{L^1({\mathbb {R}})}+ \Vert K\Vert _{L^1({\mathbb {R}})} \tfrac{C_3}{\zeta } \Big (1+ \tfrac{C_4}{c_1}\frac{ \, N/2-n }{N/2-n-C_1 k } \Big ) \Big \} . \end{aligned}$$

By choosing *C* appropriately in (40), we may assume that the parenthesis in the right-hand side above is bounded by a uniform constant. Using the previously mentioned facts that $\Vert K\Vert _{L^1({\mathbb {R}})}$ and $\zeta \Vert K'\Vert _{L^1({\mathbb {R}})}$ are bounded by a constant independent of $\zeta$, we conclude that the bracket above, when multiplied by $\zeta$, is bounded uniformly in $\zeta$. Thus, we may bound the error term of (42) as:

51 $$\begin{aligned} \frac{k}{N}\Vert f_j'\Vert _{L^1({\mathcal {I}}_k)}&\le C_5\frac{k}{N/2 -n-C_1 k}. \end{aligned}$$

Plug (47) and (51) in (41) and (42), respectively, to find that

$$\begin{aligned}A_{jj} - \sum _{\ell \ne j }A_{j\ell } \ge \frac{1}{k+1} -C_6 \frac{ k}{N/2 -n-C_1 k}.\end{aligned}$$

Taking *C* large enough in the statement of the proposition ensures that *A* is indeed diagonal dominant.

#### Remark 14

The difficulty in proving that $A_{ij}$ is diagonally dominant comes from the estimates involving *j* close to 1 or *n* as approximating sums by integrals is not efficient for these values of *j*. Another way of seeing this is that when *j* is far from 1 and *n* then $\rho (\lambda _j| {\mathfrak {q}})$ is larger and therefore the error term is smaller. Nonetheless, numerics suggest that the matrix is diagonal dominant for every $-1<\Delta < 1$ and $n\le N/2$, as shown on the plots of ${\mathbf {m}}:=\min \{A_{ii} - \sum \limits _{j \ne i} |A_{ij}|:1\le i\le n\}$ as a function of the system-size *N*, for $n=N/2$ and at four different values of $\Delta$.
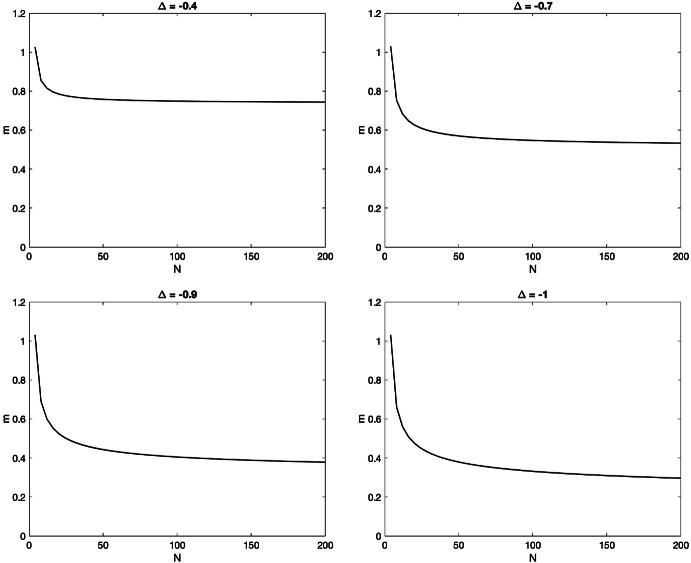


#### Remark 15

Since the differential is non-zero at $\Delta =0$ (it is diagonal since $K\equiv 0$), we obtain in particular the existence of an analytic family of solutions for *every* $n\le N/2$ for $|\Delta |\le \Delta _0$ with $\Delta _0$ small enough.

### Proof of Proposition 12 when $\Delta < -1$

Fix $\pmb \lambda$ as in the proposition. Again, $d{\mathsf {T}}_{\Delta }(\pmb \lambda )$ is not obviously positive definite. At this point, we may use a symmetrization trick like in the proof of Theorem 3 for $\Delta <-1$. This argument was presented in [14] and we refer to this paper for a full proof. Here, we present an alternative proof that we find to be of some interest.

We show that $d{\mathsf {T}}{\Delta }(\pmb \lambda )$ is invertible by estimating the large *N* behaviour of$\det [ d{\mathsf {T}}{\Delta }(\pmb \lambda )]$ with the help of Lemma 9. To start with, observe that

52 $$\begin{aligned} \widehat{\chi }(\lambda )&:= \xi (\lambda ) -\frac{1}{N} \sum _{j=1}^{n}K(\lambda - \lambda _j ) = \xi (\lambda ) - \int \limits _{-{\mathfrak {q}}}^{ {\mathfrak {q}} }K(\lambda , \mu ) \rho (\mu |{\mathfrak {q}}) d\mu \nonumber \\&\quad + O\Big ( \frac{k}{N} \Vert K^{\prime }(\lambda -\cdot ) \Vert _{ L^{1}( {\mathcal {I}}_k) } \Big ) = \rho (\lambda |{\mathfrak {q}}) \, + \, O\Big ( \frac{k}{N} \Big ) \;, \end{aligned}$$

where ${\mathcal {I}}_k:=[\Lambda \big ( 1-\frac{k}{2} |{\mathfrak {q}} \big ) , \Lambda \big ( n + \frac{k}{2} |{\mathfrak {q}} \big ) ]$ is a subinterval of $[-\pi ,\pi ]$ uniformly bounded in $n\le N /2$.

Since $\rho (\lambda |{\mathfrak {q}})\ge \tfrac{2}{\zeta }>0$ on ${\mathbb {R}}$ (by Lemma 29), the above ensures that the matrix

$$\begin{aligned} M_{ij} \, = \, \frac{ K(\lambda _i - \lambda _j) }{ \widehat{\chi }(\lambda _j) } \end{aligned}$$

is well-defined for any *n*, *N* with *N* large enough. Then, introduce an integral operator ${\mathcal {M}}$ acting on $L^2(\, (-{\mathfrak {q}}, {\mathfrak {q}} ])$ with the integral kernel

53 $$\begin{aligned} M(\lambda , \mu ) \, = \, \sum _{i,j=1}^{n} \pmb {\mathbb {1}}_{ {\mathcal {J}}_i \times {\mathcal {J}}_j }(\lambda ,\mu ) \, M_{ij} \, \rho (\mu |{\mathfrak {q}}) \end{aligned}$$

where ${\mathcal {J}}_i:=\big (\Lambda \big ( i-\tfrac{1}{2} |{\mathfrak {q}} \big ) , \Lambda \big ( i + \tfrac{1}{2} |{\mathfrak {q}} \big ) \big ]$ is a partition of the integration domain $\cup _{i=1}^{n}{\mathcal {J}}_i=(-{\mathfrak {q}}, {\mathfrak {q}} ]$ as can be inferred from the identities

$$\begin{aligned} {\mathfrak {q}}=\Lambda \big ( n+\tfrac{1}{2} |{\mathfrak {q}} \big ) = - \Lambda \big ( 1 - \tfrac{1}{2} |{\mathfrak {q}} \big ) . \end{aligned}$$

The matter is that the Fredholm determinant of ${\text {Id}}+ {\mathcal {M}}$ is equal to $\det _N[ \mathrm {I}_N + M/N]$, where $\mathrm {I}_N$ is the identity matrix. Indeed, by writing the Fredholm expansion for the determinant, one has that

$$\begin{aligned} \det _{ L^2((-{\mathfrak {q}}, {\mathfrak {q}} ]) } \big [{\text {Id}}+ {\mathcal {M}}\big ]&= \sum _{\ell =1}^{+\infty } \frac{1}{\ell !} \int \limits _{-{\mathfrak {q}}}^{ {\mathfrak {q}} } d^{\ell }\lambda \det _{\ell }\big [M(\lambda _i,\lambda _j) \big ] \\&= \sum _{\ell =1}^{+\infty } \frac{1}{\ell !} \int \limits _{-{\mathfrak {q}}}^{ {\mathfrak {q}} } d^{\ell }\lambda \sum _{ \begin{array}{c} i_1,\dots , i_{\ell }=1 \\ j_1,\dots , j_{\ell }=1 \end{array} }^{ n } \prod _{s=1}^{n} \Big \{ \pmb {\mathbb {1}}_{{\mathcal {J}}_{i_s}}(\lambda _s) \cdot \pmb {\mathbb {1}}_{{\mathcal {J}}_{j_s}}(\lambda _s) \rho (\lambda _s|{\mathfrak {q}}) \Big \}\\&\quad \times \det _{\ell }\big [M_{i_k j_u} \big ] . \end{aligned}$$

Since ${\mathcal {J}}_k\cap {\mathcal {J}}_{\ell }=\emptyset$ if $k\not =\ell$, by using that $\int \limits _{-{\mathfrak {q}}}^{ {\mathfrak {q}} } d\mu \, \pmb {\mathbb {1}}_{{\mathcal {J}}_{i}}(\mu ) \rho (\mu |{\mathfrak {q}}) \, = \, \frac{1}{N}$, one obtains

$$\begin{aligned} \det _{ L^2((-{\mathfrak {q}}, {\mathfrak {q}} ]) }\big [{\text {Id}}+ {\mathcal {M}}\big ]&= \sum _{\ell =1}^{+\infty } \frac{1}{\ell !} \sum _{ i_1,\dots , i_{\ell }=1 }^{ n } \det _{\ell }\big [ \tfrac{1}{N} M_{i_k i_u} \big ] \, = \, \det _N[\, \mathrm {I}_N + \tfrac{1}{N} M]. \end{aligned}$$

Introduce the integral operator ${\mathcal {K}}$ on $L^2((-{\mathfrak {q}}, {\mathfrak {q}} ])$ characterised by the integral kernel $K(\lambda -\mu )$. Both ${\mathcal {M}}$ and ${\mathcal {K}}$ are trace class. Indeed, ${\mathcal {M}}$ is of finite rank while ${\mathcal {K}}$ has smooth kernel and acts on functions supported on a compact interval [11]. Moreover, we have

$$\begin{aligned} \mathrm {tr}\big [ {\mathcal {K}} - {\mathcal {M}}\big ]&= \, 2 {\mathfrak {q}}K(0) - K(0) \frac{1}{N}\sum _{i=1}^{n} \frac{1}{ \widehat{\chi }(\lambda _i) } \\&= 2 {\mathfrak {q}}K(0) - K(0) \bigg \{ \int \limits _{-{\mathfrak {q}}}^{ {\mathfrak {q}} } d\mu \frac{ \rho (\mu |{\mathfrak {q}})}{ \widehat{\chi }(\mu ) } \, + \, O\Big ( \frac{k}{N} \big \Vert \frac{ \widehat{\chi }^{\, \prime } }{ \widehat{\chi }^2 } \big \Vert _{L^1({\mathcal {I}}_k)} \Big ) \bigg \} \, = \, O\Big ( \frac{k}{N} \Big ) . \end{aligned}$$

Here we used the estimate (52). We now estimate the Hilbert–Schmidt norm of ${\mathcal {K}} - {\mathcal {M}}$. One starts with the representation for the kernel

$$\begin{aligned} K(\lambda -\mu ) - M(\lambda , \mu ) \, = \, \sum _{i,j=1}^{n} \pmb {\mathbb {1}}_{{\mathcal {J}}_i\times {\mathcal {J}}_j}(\lambda ,\mu ) \, \Big \{ K(\lambda -\mu ) -K(\lambda _i - \lambda _j) \frac{ \rho (\mu |{\mathfrak {q}}) }{ \widehat{\chi }(\lambda _j) } \Big \} . \end{aligned}$$

By using that $\Lambda \big ( x+k|{\mathfrak {q}} \big )-\Lambda \big ( x |{\mathfrak {q}} \big )= O\big ( k/N \big )$ uniformly in *x*, the mean-value theorem and $(k,{\mathfrak {q}})$-interlacement of $\pmb {\lambda }$, one gets that

$$\begin{aligned} K(\lambda -\mu ) -K(\lambda _i - \lambda _j) = O\Big ( \frac{k+1}{N} \Big ) \end{aligned}$$

on ${\mathcal {J}}_i\times {\mathcal {J}}_j$. Then, the estimate (52) allows one to conclude that

$$\begin{aligned} \Big | K(\lambda -\mu ) - M(\lambda , \mu ) \Big |\, \le \, C \frac{k+1}{N} \sum _{i,j=1}^{n} \pmb {\mathbb {1}}_{{\mathcal {J}}_i\times {\mathcal {J}}_j}(\lambda ,\mu ) = C \frac{k+1}{N} . \end{aligned}$$

This yields an estimate on the Hilbert-Schmidt norm $\Vert {\mathcal {K}} - {\mathcal {M}} \Vert _{HS} = O\big ( \frac{k+1}{N} \big )$ since ${\mathfrak {q}}$ is uniformly bounded in $n\le N/2$. Thus, since both ${\mathcal {K}}, {\mathcal {M}}$ have finite Hilbert–Schmidt norms, their 2-determinants [23] satisfy

$$\begin{aligned} \det {} \! _{2}\big [{\text {Id}}+ {\mathcal {M}}\big ] - \det {} \! _{2}\big [{\text {Id}}+ {\mathcal {K}}\big ] \, = \, O\Big ( \frac{k+1}{N} \Big ) . \end{aligned}$$

Since for a trace class operator $${\mathcal {O}}$$ one has $$\det {} \! _{2}\big [{\text {Id}}+ {\mathcal {O}}\big ] = \det \! \big [{\text {Id}}+ {\mathcal {O}}\big ] \mathrm {e}^{-\mathrm {tr}[O]}$$, where the determinant appearing on the right-hand side is the usual Fredholm determinant, one infers that $$ \det \!\big [{\text {Id}}+ {\mathcal {M}}\big ] - \det \!\big [{\text {Id}}+ {\mathcal {K}}\big ] \, = \, O\big ( \frac{k+1}{N} \big ) . $$ Since $${\text {Id}}+ {\mathcal {K}}$$ is invertible on $$ L^2([-q, q ])$$ for any $$q \in [0,\pi /2]$$ (this is for instance a consequence of the proofs of Propositions 23, 26, and 28), this entails that $$\det \!\big [{\text {Id}}+ {\mathcal {M}}\big ]\not =0$$ for *N* large enough.

## Proof of Theorem 5

Let us start by stating two lemmata.

### Lemma 16

Let $\pmb \lambda (0)$ be the unique solution to (19) when $\Delta =0$. Then, one has $\Psi _N^{(n)}(\pmb \lambda (0))_{|\Delta =0}\ne 0$ and $\Lambda _N^{(n)}(\pmb \lambda (0))_{|\Delta =0}$ is the Perron-Frobenius eigenvalue of

$$\begin{aligned}V_N^{(n)}\big ( \sqrt{2} r \sin [\tfrac{\pi -\theta }{2}],\sqrt{2} r \sin [\tfrac{\theta }{2}], r \sqrt{2}\big )\end{aligned}$$

for any *r* and $\theta$.

### Proof

For $\Delta =0$, the unique solution to (19) is given by

$$\begin{aligned} \lambda _i(0) ={\mathfrak {p}}_{|\Delta =0}^{-1} \Big (2\pi \frac{i - (n+1)/2}{N}\Big ). \end{aligned}$$

It is then a matter of elementary computation to show that the entries of $\Psi _N^{(n)}$ are strictly positive, which concludes the proof of the lemma. $\square$

### Lemma 17

Let $\Delta \mapsto \pmb \lambda (\Delta )$ be an analytic solution of  (19) defined on $(-\infty ,v)$. Then, for $\Delta$ sufficiently negative, $\Psi _N^{(n)}( \pmb \lambda (\Delta ))$ is non-zero and $\Lambda _N^{(n)}( \pmb \lambda (\Delta ))$ is the Perron–Frobenius eigenvalue of $V_N^{(n)}(a,b,c)$.

### Proof of Lemma 17

First consider the special case of (19) with $\Delta =-\infty$ (see “Appendix A”) and note that in that case

$$\begin{aligned} \lambda _i(-\infty ):=\pi \frac{i - (n+1)/2}{N-n} \end{aligned}$$

for $1\le i\le n$ is the *k*-interlaced, symmetric, strictly ordered solution of (19) with $\Delta = -\infty$ (simply note that $\theta _{-\infty } (\lambda ) \equiv 2 \lambda$ in this case). Starting from (11) and (13), we deduce that if

$$\begin{aligned} \psi ^{(\infty )}(\mathbf {x}|\pmb \lambda )&:= \lim _{\Delta \rightarrow -\infty }\frac{ \psi (\mathbf {x}|\pmb \lambda ) }{ (-i\Delta )^{ \tfrac{n(n-1)}{2} }} \, = \, \prod \limits _{k=1}^{n}\mathrm {e}^{i(n+1) \lambda _{k} }\sum \limits _{\sigma \in {\mathfrak {S}}_n}{} \varepsilon (\sigma ) \prod \limits _{k=1}^{n}\mathrm {e}^{2i \lambda _{\sigma (k)}(x_k-k) } ,\\ V^{(n;\infty )}_N&:= \lim _{\Delta \rightarrow -\infty } \frac{ V_N^{(n)}(a,b,c) }{ (-2\Delta )^{ \tfrac{N}{2} -\tfrac{\theta }{\pi }(N-2n) } } ,\\ \Lambda _N^{(n;\infty )}&:= r^N, \end{aligned}$$

then the Bethe Ansatz at $\Delta =-\infty$ (or equivalently its limit as $\Delta \rightarrow -\infty$) implies that

$$\begin{aligned} V_N^{(n;\infty )}\Psi _N^{(n;\infty )} \, = \, \Lambda _N^{(n;\infty )}\Psi _N^{(n;\infty )} \qquad \text {with} \qquad \Psi _N^{(n;\infty )} := \sum _{|\mathbf {x}|=n}\psi ^{(\infty )}(\mathbf {x}|\pmb \lambda (-\infty ) )\, \varvec{\Psi }_{\mathbf {x}} . \end{aligned}$$

One gets the result by proving that $\Psi _N^{(n;\infty )}$ is non-zero and that $\Lambda _N^{(n;\infty )}$ is the largest eigenvalue of $V_N^{(n;\infty )}$, which we next do.

We start by showing the first claim by considering the entry of $\Psi _N^{(n;\infty )}$ for $\mathbf {x}_{{\mathfrak {e}}}=(2,4,\dots ,2n)$. Setting $\tau = \mathrm {e}^{2\pi i/(N-n)}$, one deduces from the above that

$$\begin{aligned} \psi ^{(\infty )}(\mathbf {x}_{{\mathfrak {e}}}|\pmb \lambda (-\infty ) ) \; = \; \tau ^{ -\tfrac{n}{4}(n+1)^2 } \det \big [\tau ^{j\cdot k}\big ]_{j,k} . \end{aligned}$$

The determinant of the Vandermonde matrix $(\tau ^{j\cdot k})_{j,k}$ does not vanish since it corresponds to the values $\tau , \tau ^2, \dots , \tau ^n$, which are all distinct owing to $2n \le N$. Hence, $\Psi _N^{(n;\infty )} \ne 0$.

For the second property, we refer to [14, Lemma 3.2] for the full proof. $\square$

Finally, we are ready to prove the theorem.

### Proof of Theorem 5

Consider an analytic family of solutions $\Delta \mapsto \pmb \lambda (\Delta )$ as in the statement of the theorem. Then $\Delta \mapsto \Psi _N^{(n)}(\pmb \lambda (\Delta ))$ is an analytic family of vectors. By Lemma 16 for $(u,v) = (\Delta _0, 1)$ or Lemma 17 for $(u,v) = (-\infty ,\Delta _0)$, $\Psi _N^{(n)}(\pmb \lambda (\Delta _0))\ne 0$ for some $\Delta _0\in (u,v)$. The analyticity implies that $\Psi _N^{(n)}( \pmb \lambda (\Delta ))\ne 0$ for all but a discrete set *D* of $\Delta$ in (*u*, *v*).

It follows that $\Lambda _N^{(n)}( \pmb \lambda (\Delta ))$ is an eigenvalue of $V_N^{(n)}(a,b,c)$ for all $\Delta \in (u,v){\setminus } D$. By continuity of $(a,b,c)\mapsto V_N^{(n)}(a,b,c)$ and $\Delta \mapsto \Lambda _N^{(n)}(\pmb \lambda (\Delta ))$, this property extends to all values $\Delta \in (u,v)$.

Now, since $V_N^{(n)}(a,b,c)$ is an irreducible symmetric matrix, its Perron-Frobenius eigenvalue is isolated for all *a*, *b*, *c* with $\Delta \in (u,v)$. Lemmata 16 and 17 proved that $\Lambda _N^{(n)}( \pmb \lambda (\Delta ))$ is the Perron-Frobenius eigenvalue for *some* $\Delta \in (u,v)$; by the fact that the region of *a*, *b*, *c* values with $\Delta \in (u,v)$ is connected, and by the continuity of both $(a,b,c)\mapsto V_N^{(n)}(a,b,c)$ and $(a,b,c)\mapsto \Lambda _N^{(n)}$ this property extends to the whole set of parameters *a*, *b*, *c* with $\Delta \in (u,v)$. $\square$

### Remark 18

The analyticity of $\Delta \mapsto \pmb \lambda (\Delta )$ was only used once in the proof above, namely to show that the vector $\Psi _N^{(n)}( \pmb \lambda (\Delta ) )$ is non-zero for (almost) all $\Delta$.

It is non-trivial that this property holds for *all* $\Delta$, *N* and *n*. The norm of $\Psi _N^{(n)}( \pmb \lambda (\Delta ) )$ has been argued to be given in terms of the determinant of $d{\mathsf {T}}_\Delta (\pmb \lambda )$ in [20, 27] and this was proven in [26, 37]. The results reads

54 $$\begin{aligned} \Vert \Psi _N^{(n)}( \pmb \lambda (\Delta ) )\Vert ^2=f(\pmb \lambda )\det [d{\mathsf {T}}_\Delta (\pmb \lambda )] \end{aligned}$$

for some explicit non-zero function *f*. Therefore, proving that the vector is non-zero amounts to proving that the differential of ${\mathsf {T}}_\Delta$ is invertible which, as shown above, automatically implies analyticity.

In conclusion, proving analyticity of the solutions and using the strategy above, rather than proving their continuity and separately that the resulting vector is non-zero, bypasses the use of (54) and contains no additional complications.

## Proof of Theorem 6

Let $k=C_0\log N$ and $n\le N/2-C_1k^2$. The constants $C_0$ and $C_1$ will be chosen large enough in the course of the proof.

To start, we follow the argument of Theorem 3 in Sect. 2.2 to guarantee that for each $-1<\Delta <0$, every $(k, {\mathfrak {q}})$-interlaced solution is strictly interlaced. For the proof to work, we need to check that

55 $$\begin{aligned} |\vartheta (\lambda _i-\lambda _1)-\vartheta (\lambda _i-{\mathfrak {q}})|\le 2\pi \tfrac{k}{k+1}. \end{aligned}$$

In order to do that, remark that the value of the extremum of $\vartheta$ and the $(k,{\mathfrak {q}})$-interlacement imply that

$$\begin{aligned} |\vartheta (\lambda _i-\lambda _1)-\vartheta (\lambda _i-{\mathfrak {q}})| \le 2|\vartheta \big (\tfrac{\lambda _1-{\mathfrak {q}}}{2}\big )| \end{aligned}$$

owing that the maximum of the difference is attained at the midpoint. Then, by using interlacement

$$\begin{aligned} |\vartheta (\lambda _i-\lambda _1)-\vartheta (\lambda _i-{\mathfrak {q}})| \le 2|\vartheta \big (\Lambda (1-\tfrac{k}{2}|{\mathfrak {q}})\big )| . \end{aligned}$$

Further, upon using the explicit expression for $\vartheta$ given in “Appendix A”, one gets

$$\begin{aligned} |\vartheta (\lambda _i-\lambda _1)-\vartheta (\lambda _i-{\mathfrak {q}})|&\le 4 \big |\arctan \big [\tanh \big (\Lambda (1-\tfrac{k}{2}|{\mathfrak {q}}\big )\cot (\zeta ) \big ] \big | \\&\le 2\pi - 4 \arctan \bigg [ \frac{\zeta }{ \Lambda \big (1-\tfrac{k}{2} | Q( \tfrac{1}{2} - C_1 \tfrac{k^2}{N} ) \big ) } \bigg ] . \end{aligned}$$

The last inequality above follows via simple trigonometric manipulations from $\tanh (y)\le y$, $\cot (\zeta )\le 1/\zeta$ and the fact that $q \mapsto \Lambda (x|q)$ is decreasing for $x< (n+1)/2$ in this regime of $\Delta$. The latter is easily inferred by taking the *q* derivative of the equation defining $\Lambda (x|q)$. Using (46), the above then leads to

$$\begin{aligned} |\vartheta (\lambda _i-\lambda _1)-\vartheta (\lambda _i-{\mathfrak {q}})|&\le 2\pi - \frac{4\zeta }{\Lambda \big (1-\tfrac{k}{2}|Q( \tfrac{1}{2} - C_1 \tfrac{k^2}{N} ) \big ) } \\&\le 2\pi - \frac{ C }{\log \big [ \frac{C^{\prime }}{N}(C_1 k^2-C^{\prime \prime }k) \big ]} . \end{aligned}$$

Overall, we deduce that there exits a constant $c_3>0$ independent of everything such that for every $-1<\Delta <0$,

$$\begin{aligned} |\vartheta (\lambda _i-\lambda _1)-\vartheta (\lambda _i-{\mathfrak {q}}) | \le 2\pi - \frac{c_3}{\log N}. \end{aligned}$$

We deduce (55) by fixing $C_0$ large enough. In particular, we obtain the equivalent of Theorem 3, namely that for every $-1<\Delta <0$, there exists a $(k,{\mathfrak {q}})$-interlaced strictly ordered symmetric solution $\pmb \lambda (\Delta )$ to (19).

We now need to prove that the family $\pmb \lambda (\Delta )$ can be assumed to be analytic. We follow the argument of Sect. 3.2, with minor changes which we describe next. As in Sect. 3.2 we may use (55) to deduce (41) and (42). It remains to bound the error terms. We start with (42). Since $N/2-n-k\ge C_1k^2-k$, (51) leads to

$$\begin{aligned} \frac{k}{N}\Vert f_j'\Vert _{L^1( {\mathcal {I}}_k) }\le \frac{k|2\pi -4\zeta |}{c_0c_3(N/2-n-Ck)}\le \frac{C_4}{C_1k-1}. \end{aligned}$$

This can be made smaller than 1/(4*k*) by choosing *C* large enough. The same argument applies to the error term in (41) and the proof follows.

### Remark 19

The uniform $(C_0\log N,{\mathfrak {q}})$-interlacement shows that the entries of $\tfrac{1}{\zeta }\pmb \lambda (\Delta )$ are bounded uniformly in $\Delta$ so that we may extract sub-sequential limits as $\zeta$ tends to 0 to approach $(C_0\log N,{\mathfrak {q}})$-interlaced strictly ordered symmetric solutions $\pmb \lambda (-1)$ to (19) for $\Delta =-1$. Here, one should use the convergence on compact subsets of ${\mathbb {R}}$, as $\Delta \rightarrow -1$, *viz*. $\zeta \rightarrow 0^+$, of ${\mathfrak {p}}(\cdot /\zeta )$, $\vartheta (\cdot /\zeta )$, $\tfrac{1}{\zeta } K(\cdot /\zeta )$ and $\tfrac{1}{\zeta } \xi (\cdot /\zeta )$ to ${\mathfrak {p}}_{|\Delta =-1}$, $\vartheta _{|\Delta =-1}$, $K_{|\Delta =-1}$ and $\xi _{|\Delta =-1}$ given in “Appendix A.2”.

To prove an analogue of Theorem 3 for $\Delta =-1$, one may employ the bounds of Sect. 2.2 and rescale all variables by $1/\zeta$. This allows one to conclude that $\Psi$ maps $\tfrac{1}{\zeta } {\bar{\Omega }}_{k,R}$ onto $\tfrac{1}{\zeta } \Omega _{k,R}$ and so, by taking the $\zeta \rightarrow 0$ limit, yields a Brouwer fixed point for the map $\Psi _{|\Delta =-1}$. The unique fixed point of this map coincides, by construction, with any sub-sequential limit of $\tfrac{1}{\zeta }\pmb \lambda (\Delta )$ as $\zeta$ tends to 0. Thus, such sub-sequential limits are unique, which shows that $\tfrac{1}{\zeta }\pmb \lambda (\Delta )$ does converge to a $(C_0\log N,{\mathfrak {q}})$-interlaced, strictly ordered, symmetric solution $\pmb \lambda (-1)$ to (19) for $\Delta =-1$.

## Proof of Theorem 1

We prove the statement for $a> b$ and $\Delta \ne -1$. The results extends to all $a\ge b\ge 0$ and $c\ge 0$ with $\Delta <1$, since the left and right sides of (6) are Lipschitz in each coordinate of (*a*, *b*, *c*). The particular expression for $\Delta = -1$ is obtained by taking the limit either from above or below in (6).

We start with the expression

$$\begin{aligned} Z({\mathbb {T}}_{N,M},a,b,c)=\sum _{n=0}^{N}Z^{(n)}({\mathbb {T}}_{N,M},a,b,c), \end{aligned}$$

which gives

56 $$\begin{aligned}&\lim _{M\rightarrow \infty }\tfrac{1}{NM}\log Z({\mathbb {T}}_{N,M},a,b,c)=\max _{n\le N/2}f_N^{(n)}(a,b,c)=f_N^{(N/2)}(a,b,c), \end{aligned}$$

where in the first equality we restrict our attention to $n\le N/2$ thanks to the symmetry $n\longleftrightarrow N-n$ corresponding to the symmetry under the reversal of all arrows. The last equality uses the classical fact that $f_N^{(n)}(a,b,c)$ is maximal for $n=N/2$, see [14, Lemma 3.6] or [31] for a proof.

For $0\le \Delta < 1$ or $\Delta < -1$, we apply (22) and evaluate (14) at the $n=N/2$ groundstate’s Bethe roots to get

57 $$\begin{aligned} f_N^{(N/2)}(a,b,c)= \text {max} \Big \{&\log a+\int \limits _{-Q(1/2)}^{Q(1/2)} \log |L( \lambda ) | \rho (\lambda ) d\lambda +O(\tfrac{1}{N}) \;, \nonumber \\&\quad \log b + \int \limits _{-Q(1/2)}^{Q(1/2)} \log |M( \lambda ) | \rho (\lambda ) d\lambda +O(\tfrac{1}{N}) \Big \} . \end{aligned}$$

We claim that the same holds for $-1<\Delta <0$. Notice however that we do not have access to $f_N^{(N/2)}(a,b,c)$ in this case. However, the inequality^7^

$$\begin{aligned} Z^{(n)}({\mathbb {T}}_{N,M},a,b,c)\ge \frac{1}{N}\Big (\frac{\min \{a,b,c\}}{4\max \{a,b,c\}}\Big )^{MN/n}Z^{(n+1)}({\mathbb {T}}_{N,M},a,b,c) \end{aligned}$$

is sufficient to obtain (57) as we can apply (22) to $$n:=N/2-C_0$$, and then compare $$ f_N^{(N/2)}$$ to $$f_N^{(n)}$$ and $$\rho (\lambda )$$ to $$\rho (\lambda |Q(\tfrac{1}{2}-\tfrac{C_0}{N}))$$.

Overall, we see that the only remaining difficulty is to compute

$$\begin{aligned} \log a + \int \limits _{-Q(1/2)}^{Q(1/2)} \log |L( \lambda ) |\rho (\lambda )d\lambda \quad \text {and} \quad \log b + \int \limits _{-Q(1/2)}^{Q(1/2)} \log |M( \lambda ) | \rho (\lambda ) d\lambda . \end{aligned}$$

We shall only focus on the evaluation of the first term and split the proof in two depending on whether $|\Delta |<1$ or $\Delta < -1$. We leave to the reader the verification that the first term does indeed dominate the second when $a>b$.

### Case $|\Delta |<1$

When $|\Delta |<1$, we use the Fourier transform on ${\mathbb {R}}$. Define

58 $$\begin{aligned} {\mathscr {L}}(x):=\tfrac{1}{2}\log [L(x)L(-x)] = \log |L(x)| \qquad \hbox { for} x \in {\mathbb {R}} \end{aligned}$$

and observe the exact expressions given in “Appendix A” for the different functions and their Fourier transforms. Recalling that $\rho$ is even and $Q(1/2)=+\infty$, we find that

$$\begin{aligned} f(a,b,c)-\log a&=\int \limits _{-\infty }^{+\infty } {\mathscr {L}}(x)\rho (x)dx=\tfrac{1}{2\pi }\int \limits _{-\infty }^{+\infty }\widehat{{\mathscr {L}}}(t)\widehat{\rho }(t)dt\\&= \int \limits _{-\infty }^{+\infty }\frac{1}{2\cosh {(\zeta \frac{t}{2})}}\frac{ \sinh (\frac{\theta \zeta }{\pi }t)}{t}\frac{\sinh [(\pi - \zeta ) \frac{t}{2}]}{\sinh {[\pi \frac{t}{2}]}}dt. \end{aligned}$$

Using that

$$\begin{aligned} \log \frac{b}{a} \; = \; \int \limits _{-\infty }^{+\infty } \frac{\sinh \big [ t\zeta (\tfrac{\theta }{\pi }-\tfrac{1}{2})\big ] \sinh \big [ (\pi - \zeta ) \tfrac{t}{2} \big ] }{ t \sinh {\big [ \pi \tfrac{t}{2} \big ] }}d t \end{aligned}$$

some algebra and the change of variables $t\mapsto 2t$ give the result.

#### Remark 20

For the special case $a=b=c=1$, we may compute the integral directly. After a fairly elementary computation, we recover the classical result of Lieb [31]

$$\begin{aligned} f(1,1,1)&=\int \limits _{-\infty }^{\infty } \frac{\tfrac{1}{2}\log [1-\frac{3}{1+2\cosh x}]}{\tfrac{8\pi }{3}\cosh (3x/4)}dx=\tfrac{3}{2}\log [\tfrac{4}{3}]. \end{aligned}$$

The particular expression for $a= b= 1$ and $c=2$ is obtained from (6) by direct computation.

### Case $\Delta < -1$

When $\Delta < -1$, we work with $\pi$-periodic functions and consider Fourier coefficients. Again, we introduce ${\mathscr {L}}(x):=\tfrac{1}{2}\log [L(x)L(-x)]$ and use the exact expression of the functions and their Fourier coefficients given in “Appendix A”. We deduce that

59 $$\begin{aligned} \begin{aligned} f(a,b,c)-\log a&=\int \limits _{-\pi /2}^{\pi /2} {\mathscr {L}}(x)\rho (x)dx=\tfrac{1}{\pi }\sum _{n=0}^\infty {\widehat{\rho }}(n)\widehat{ {\mathscr {L}}}(n) \\&= \frac{\theta \zeta }{\pi } + \sum _{n\in {\mathbb {Z}}{\setminus }\{0\}} \mathrm {e}^{-|n|\zeta } \frac{ \sinh \big [ 2n \zeta \theta /\pi \big ]}{ 2 n \cosh (\zeta n) }. \end{aligned} \end{aligned}$$

## Proof of Theorem 2

### Focusing on the asymptotic in the *q* variable

We claim that it suffices to estimate the asymptotic behaviour of

60 $$\begin{aligned} \delta f(q) :=\int \limits _{ - Q(1/2) }^{ Q(1/2) } {\mathscr {L}}(x)\rho (x)dx - \int \limits _{-q}^{q} {\mathscr {L}}(x)\rho (x|q)dx \end{aligned}$$

as $q\nearrow Q(1/2)$, with ${\mathscr {L}}(x)$ defined in (58).

Indeed, (22) shows that $f(a,b,c) -f^{(n)}(a,b,c) = \delta f(Q(n/N)) + O(1/N)$. Recall that (22) is obtained from Theorem 4 whenever $\Delta \ne -1$ and (7) holds. For $\Delta = -1$, if (7) is satisfied, (22) may be deduced from Theorem 6.

The asymptotics of *Q*(*n*/*N*) as *n*/*N* approaches 1/2 are given by Propositions 25, 27 and Lemma 30 of “Appendixes B, C and D”, respectively, and read

$$\begin{aligned} \lim _{m\rightarrow 1/2}( \tfrac{1}{2}-m) \mathrm {e}^{Q(m)\tfrac{\pi }{\zeta }}&=:C_\Delta \hbox { for} \Delta \in [-1,1) \quad \text {and} \lim _{m\rightarrow 1/2}\frac{ 1 - 2m }{ \pi -2 Q(m) }\\&=\rho (\tfrac{\pi }{2}) \hbox { for} \Delta <-1 \end{aligned}$$

for some constant $C_{\Delta }>0$.

The estimation of (60) is obtained differently for $-1 \le \Delta <1$ and $\Delta < -1$ and does not refer anymore to the discrete Bethe equation. Deriving Theorem 2 from the asymptotics of (60) obtained below and those for *Q* mentioned above is a matter of simple algebra, which we do not detail further.

#### Remark 21

The constant $C_\Delta$ was explicitly computed in [12]. We do not need the precise value here and therefore work with this weaker and simpler result. We provide however in Proposition 25 of “Appendix B” an integral representation for $C_{\Delta }$ in terms of the solution to a Wiener-Hopf equation on ${\mathbb {R}}_+$.

### Case $|\Delta |<1$

Consider the function *G* defined for $x\in {\mathbb {R}}$ by

$$\begin{aligned} G(x):={\mathscr {L}}(x)-\int \limits _{{\mathbb {R}}}R(x-y){\mathscr {L}}(y)dy. \end{aligned}$$

Using (23), then reorganizing the integrals (in particular using that ${\mathscr {L}}$ and *R* are even), and then passing to Fourier gives that

$$\begin{aligned} \delta f(q)&=\int \limits _{{\mathbb {R}}}{\mathscr {L}}(x)\rho (x|q) \pmb {\mathbb {1}}_{|x|>q}dx-\int \limits _{{\mathbb {R}}}\int \limits _{{\mathbb {R}}}{\mathscr {L}}(x)R(x-y)\rho (y|q)\pmb {\mathbb {1}}_{|y|>q}dxdy \\&=\int \limits _{{\mathbb {R}}}G(x)\rho (x|q)\pmb {\mathbb {1}}_{|x|>q}dx\\&=\int \limits _{{\mathbb {R}}} {\widehat{G}}(t)\widehat{\rho (\cdot |q)}(t)\frac{dt}{2\pi }-\int \limits _{{\mathbb {R}}}\int \limits _{{\mathbb {R}}} {\widehat{G}}(t)\frac{ \mathrm {e}^{iq(t-s)} - \mathrm {e}^{iq(s-t)}}{i(t-s)}\widehat{\rho (\cdot |q)}(s)\frac{ds}{2\pi }\frac{dt}{2\pi } \\&=\int \limits _{{\mathbb {R}}} {\widehat{G}}(t)\widehat{\rho (\cdot |q)}(t)\frac{dt}{2\pi } -\lim _{\delta \searrow 0}\int \limits _{{\mathbb {R}}+i\delta }\Big (\int \limits _{{\mathbb {R}}} {\widehat{G}}(t)\frac{ \mathrm {e}^{iq(t-s)} - \mathrm {e}^{iq(s-t)}}{i(t-s)}\frac{dt}{2\pi }\Big ) \widehat{\rho (\cdot |q)}(s)\frac{ds}{2\pi } . \end{aligned}$$

In the last identity we use that

$$\begin{aligned} {\widehat{G}}(k) = \frac{ \widehat{{\mathscr {L}}}(k) }{1+{\widehat{K}}(k)} \, = \, \frac{\pi }{k} \frac{ \sinh \big [ k\zeta \tfrac{\theta }{\pi } \big ] }{ \cosh \big [ k\tfrac{\zeta }{2} \big ] } \end{aligned}$$

is integrable on a neighbourhood of ${\mathbb {R}}$ and has exponential decay. We now perform two elementary residue computations. Fix $\pi /\zeta<\beta <3\pi /\zeta$ and $s\in i\delta +{\mathbb {R}}$ for $\delta >0$ very small. Since ${\text {Res}}_{t=\pm i\pi /\zeta }[{\widehat{G}}]={\mp } 2i\sin \theta$ and there is no other pole of ${\widehat{G}}$ in the strip $\{z\in {\mathbb {C}}:\mathrm{Im}(z)<\beta \}$ (and ${\widehat{G}}$ tends to 0 at infinity), we get

$$\begin{aligned} \int \limits _{\mathbb {R}} \frac{\mathrm {e}^{iq(t-s)}}{t-s} \widehat{G}(t)\frac{dt}{2 i \pi }&= \widehat{G}(s) + \frac{\mathrm {e}^{iq(i\pi /\zeta -s)}}{i\pi /\zeta - s}{\text {Res}}_{t= \pm i\pi /\zeta }[\widehat{G}] + \int \limits _{{\mathbb {R}}+\beta i} \frac{ \mathrm {e}^{iq(t-s)} }{i(t-s)} \widehat{G}(t) \frac{dt}{2\pi } \\ {}&= \widehat{G}(s)-2i\sin \theta \,\frac{\mathrm {e}^{-q\pi /\zeta -iqs}}{i\pi /\zeta - s} + \mathrm {e}^{-\beta q } \psi _{q}^{(+)}(s), \end{aligned}$$

where

61 $$\begin{aligned} \psi _{q}^{(\pm )}(s) \, = \, \pm \int \limits _{{\mathbb {R}} } \frac{ \mathrm {e}^{ \pm iq(t-s)} \widehat{G}(t \pm i \beta ) }{t-s \pm i \beta } \frac{dt}{2 i \pi } \end{aligned}$$

and similarly

$$\begin{aligned} -\int \limits _{\mathbb {R}} \frac{ \mathrm {e}^{iq(s-t)} }{ t-s } \widehat{G}(t)\frac{dt}{2 i \pi }&=-2i\sin \theta \,\frac{\mathrm {e}^{-q\pi /\zeta +iqs}}{i\pi /\zeta +s} + \mathrm {e}^{-\beta q } \psi _{q}^{(-)}(s). \end{aligned}$$

Putting these two displayed equations in the first one gives that

62 $$\begin{aligned} \begin{aligned} \delta f(q) =&\, 2i \sin \theta \, \mathrm {e}^{-\frac{q\pi }{\zeta } }\int \limits _{\mathbb {R}}\widehat{\rho (\cdot |q)}(s)\left( \frac{\mathrm {e}^{-iqs}}{ i\pi /\zeta -s}+\frac{ \mathrm {e}^{iqs}}{i\pi /\zeta +s}\right) \frac{ds}{2\pi } \\&- \mathrm {e}^{-\beta q } \int \limits _{\mathbb {R}} \widehat{\rho (\cdot |q)}(s)( \psi _{q}^{(+)}(s) + \psi _{q}^{(-)}(s) )ds . \end{aligned} \end{aligned}$$

We first justify that the second term is a $O(\mathrm {e}^{-\beta q })$. Clearly, $\psi _{q}^{(\pm )} \in L^2({\mathbb {R}})$ and $\Vert \psi _q^{(\pm )}\Vert _{L^2({\mathbb {R}})}\le C$ uniformly in *q*. Furthermore, it is established in Proposition 25 of “Appendix B” that given

$$\begin{aligned} {\mathfrak {e}}(x) := \frac{1}{\zeta }\mathrm {e}^{-\tfrac{\pi }{\zeta }x} \pmb {\mathbb {1}}_{{\mathbb {R}}_+}(x), \end{aligned}$$

we find that

63 $$\begin{aligned} \rho (x|q)=\rho (x) \, + \, \mathrm {e}^{-\frac{q\pi }{\zeta }} \big [ (T-{\mathfrak {e}})(x-q)+(T-{\mathfrak {e}})(-q-x)+\delta T(x) \big ] \end{aligned}$$

where $\Vert \delta T\Vert _{L^{\infty }({\mathbb {R}})}+\Vert \delta T\Vert _{L^1({\mathbb {R}})} \, = \, O(e^{-2q})$ and *T* is the unique solution of the integral equation

64 $$\begin{aligned} T(x) -\int \limits _0^\infty R(x-y)T(y)dy ={\mathfrak {e}}(x) . \end{aligned}$$

By interpolation theorems for $L^p$ spaces, we get that *T* and $\delta T$ belong to $L^2({\mathbb {R}})$ with norms uniformly bounded in *q*. Therefore, $\rho ( \cdot |q) \in L^2({\mathbb {R}})$ with a norm controlled uniformly in *q*. All of this ensures that the last term in (62) is indeed $O(\mathrm {e}^{-\beta q })$.

Since $\widehat{\rho (\cdot |q)}$ is even and $\tfrac{1}{\pi /\zeta +is}$ is the Fourier transform of ${\mathfrak {e}}$, we find

65 $$\begin{aligned} \int \limits _{\mathbb {R}}\widehat{\rho (\cdot |q)}(s)\left( \frac{\mathrm {e}^{-iqs}}{\pi /\zeta +is}+\frac{\mathrm {e}^{iqs}}{\pi /\zeta -is}\right) \frac{ds}{2\pi }&=2 \int \limits _0^\infty \rho (x+q|q) \mathrm {e}^{-x \frac{\pi }{\zeta } } dx. \end{aligned}$$

Plugging $x+q$ in (63), and performing an asymptotic expansion of (63) (at first order) enables us to recast (62) as

$$\begin{aligned} \delta f(q)&= 4 \sin \theta \, \mathrm {e}^{-q \frac{\pi }{\zeta } } \int \limits _0^\infty \mathrm {e}^{-x \frac{\pi }{\zeta } } \Big \{ \rho (x+q) + \mathrm {e}^{-q \frac{\pi }{\zeta } } \big [ T(x) - {\mathfrak {e}}(x) \\&\quad + T(-x-2q) + \delta T(x+q) \big ] \Big \}dx + o\big ( \mathrm {e}^{-2q \frac{\pi }{\zeta } } \big ), \end{aligned}$$

where we used the fact that $\beta$ can be taken strictly larger than $2\pi /\zeta$. Then, by using

- $\rho (x+q)={\mathfrak {e}}(x+q) \big ( 1 + \mathrm {e}^{-\frac{ 2\pi }{\zeta } q} \delta {\mathfrak {e}}(x) \big )$ with $\Vert \delta {\mathfrak {e}} \Vert _{L^1({\mathbb {R}}_+)}$ bounded uniformly in *q*,
- $$\delta T\in L^{\infty }({\mathbb {R}})$$,
- the fact that as $\lambda$ tends to infinity,
  $$\begin{aligned}T(\lambda )=O\big ( \max \big \{ \mathrm {e}^{ - \frac{2\pi }{\pi -\zeta } \lambda }, \mathrm {e}^{ - \frac{\pi }{\zeta } \lambda } \big \} \big ).\end{aligned}$$
  which follows from the integral equation satisfied by *T*, the fact that $T\in L^1({\mathbb {R}})$ and similar estimates for the behaviour of $R(\lambda )$ when $\lambda \rightarrow \pm \infty$,

one readily infers that

$$\begin{aligned} \delta f(q)&= 4 \sin \theta \, \mathrm {e}^{-2q \frac{\pi }{\zeta } } \cdot \int \limits _0^\infty \mathrm {e}^{-x \tfrac{\pi }{\zeta }} T(x)dx \; + \; o\big ( \mathrm {e}^{-2q \frac{\pi }{\zeta } } ). \end{aligned}$$

Thus, all-in-all, we get that with ${\mathfrak {q}}=Q(n/N)$,

$$\begin{aligned} \delta f({\mathfrak {q}})&= ( 1 + o(1) ) \big ( \tfrac{1}{2}-\tfrac{n}{N} \big )^2 \sin \theta \cdot \tfrac{4}{C_{\Delta }^2 } \int \limits _0^\infty \mathrm {e}^{-x \tfrac{\pi }{\zeta }} T(x)dx . \end{aligned}$$

Note that the constant is strictly positive since $T>0$ on ${\mathbb {R}}$.

### Case $\Delta = -1$

We omit the proof as it is the same as in the previous section, using “Appendix C” instead of “Appendix B”.

### Case $\Delta < -1$

Following the same reasoning as in the previous section gives

$$\begin{aligned} \delta f(q):=&\int \limits _{-\pi /2}^{\pi /2} {\mathscr {L}}(x)\rho (x)dx - \int \limits _{-q}^q {\mathscr {L}}(x)\rho (x|q)dx\\ =&(\pi -2q)\rho (\tfrac{\pi }{2})\big [{\mathscr {L}}(\tfrac{\pi }{2}) - \int \limits _{-\pi /2}^{\pi /2} {\mathscr {L}}(x)R(x-\tfrac{\pi }{2})dx+o(1)\big ]. \end{aligned}$$

Above, the *o*(1) term is as $q\rightarrow \pi /2$. It remains to prove that the following quantity is strictly positive:

$$\begin{aligned} {\mathscr {L}}(\tfrac{\pi }{2}) - \int \limits _{-\pi /2}^{\pi /2} {\mathscr {L}}(x)R(x-\tfrac{\pi }{2})dx&=\frac{1}{\pi }\sum _{n\in {\mathbb {Z}}} (-1)^n \widehat{{\mathscr {L}}}(n) - \frac{1}{\pi } \sum _{n\in {\mathbb {Z}}} (-1)^n \widehat{{\mathscr {L}}}(n)\widehat{R}(n)\\&= \frac{1}{\pi } \sum _{n\in {\mathbb {Z}}} (-1)^n\frac{\widehat{{\mathscr {L}}}(n)}{1+ \widehat{K}(n)}\\&= \sum _{n\in {\mathbb {Z}}} (-1)^n\frac{ \sinh \big [ 2n \zeta \tfrac{\theta }{\pi } \big ] }{ 2n \cosh (n\zeta ) }. \end{aligned}$$

The above is the convolution of the inverse Fourier transforms of $\tanh [n\zeta ]/(2n)$ and $\sinh {[2n\zeta \tfrac{\theta }{\pi }]}/\sinh {[n\zeta ]}$ evaluated at $\pi /2$. Both of these inverse Fourier transforms may be shown to be positive using Poisson summation, which in turn implies the positivity of the above expression.

## A Refined Version of Theorem 2 (Under Additional Conditions)

In this section, we prove a sharper version of Theorem 2 under mild conditions on *a*, *b*, *c* and *n*, *N*.

### Theorem 22

For $N\ge 2$ and $a\ne b$ and $c\ge 0$ leading to $|\Delta |<1$, there exists a constant $C=C(\zeta )<\infty$ such that for every $n\le \tfrac{1}{2}N-C/\zeta$,

66 $$\begin{aligned} f_N^{(n)}(a,b,c)= f(a,b,c)- C(\zeta )\sin \theta (1+o(1))(1-\tfrac{2n}{N})^2+O(\tfrac{1}{\zeta (N-2n)N}),\qquad \end{aligned}$$

where *o*(1) is a quantity tending to zero as *n*/*N* tends to 1/2.

The improvement with respect to Theorem 2 is that the *O*(1/*N*) is replaced with the more precise $O(\tfrac{1}{\zeta (N-2n)N})$. This will be particularly useful in [17], where it is used to prove that $N|f_N^{(n)}(a,b,c) - f_N^{(n+1)}(a,b,c)| \rightarrow 0$ as $N \rightarrow \infty$, with $N-2n \le \sqrt{N}$. This convergence is expected to hold for all $n = N/2 + o(N)$, when $\Delta \in [-1,1)$.

### Proof of Theorem 22

Due to the form of (66), it suffices to prove the statement for *N* large enough. Fix for now *n* and *N* as in the theorem; we will see later which bounds are needed on *N*.

Consider the analytic family $\pmb \lambda =(\lambda _i:1\le i\le n)$ given by Theorem 4 on $(\Delta _0, 1)$. We start by improving on the condensation formula of Theorem 3. Introduce, for a fixed $\Delta$, the quantity ${\text {Diff}}: [1,\dots , n]\rightarrow {\mathbb {R}}$ defined by

$$\begin{aligned} {\text {Diff}}(i)&:= N\int _{\Lambda (i|{\mathfrak {q}})}^{\lambda _i} \rho (\lambda |{\mathfrak {q}})\, d\lambda ,\\ {\text {Diff}}&:=\max \{|{\text {Diff}}(i)|:1\le i\le n\}. \end{aligned}$$

### Claim 1

There exists $C_0>0$ such that for every $f:{\mathbb {R}} \rightarrow {\mathbb {R}}$ with integrable first and second derivatives, and for every $\pmb \lambda =(\lambda _i:1\le i\le n)$ with $\mathrm {Diff}\le \tfrac{1}{2}$,

$$\begin{aligned}&\Big |\frac{1}{N} \sum _{j = 1}^n f(\lambda _j) - \int _{-{\mathfrak {q}}}^{{\mathfrak {q}}} f(\lambda ) \rho (\lambda | {\mathfrak {q}})\, d\lambda \Big |\\&\quad \le \frac{\mathrm {Diff}}{N}\Vert f'\Vert _{L^1[-{\mathfrak {q}},{\mathfrak {q}}]}+\frac{C_0(1+\mathrm {Diff})}{N(N-2n-C_0)} (\zeta \Vert f''\Vert _{L^1[-{\mathfrak {q}},{\mathfrak {q}}]}+\Vert f'\Vert _{L^1[-{\mathfrak {q}},{\mathfrak {q}}]}). \end{aligned}$$

### Proof

Using that the integral of $\rho (\lambda |{\mathfrak {q}})$ between $\Lambda (j-\tfrac{1}{2}|{\mathfrak {q}})$ and $\Lambda (j+\tfrac{1}{2}|{\mathfrak {q}})$ is $\tfrac{1}{N}$ gives

$$\begin{aligned} \frac{1}{N} \sum _{j = 1}^n f(\lambda _j)-\int _{-{\mathfrak {q}}}^{\mathfrak {q}} f(\lambda )\rho (\lambda |{\mathfrak {q}})d\lambda&= \sum _{j = 1}^{n} \int _{\Lambda (j-1/2|{\mathfrak {q}})}^{\Lambda (j+1/2|{\mathfrak {q}})}(f(\lambda _j)-f(\lambda ))\rho (\lambda |{\mathfrak {q}})d\lambda . \end{aligned}$$

Differentiating the definition of $\Lambda (x|{\mathfrak {q}})$ gives

$$\begin{aligned} \Lambda '(y|{\mathfrak {q}})=\frac{1}{N\rho (\Lambda (y|{\mathfrak {q}})|{\mathfrak {q}})}. \end{aligned}$$

Therefore, if we set $I_j:=[j-\tfrac{1}{2},j+\tfrac{1}{2}]$ and $g(y):=f(\Lambda (y|{\mathfrak {q}}))$, a change of variables implies, for every *j*,

$$\begin{aligned} \Big |\int _{\Lambda (j-1/2|{\mathfrak {q}})}^{\Lambda (j+1/2|{\mathfrak {q}})}(f(\Lambda (j|{\mathfrak {q}}))-f(\lambda ))\rho (\lambda |{\mathfrak {q}})d\lambda \Big |&=\frac{1}{N}\Big |\int _{j-1/2}^{j+1/2}(g(j)-g(x))dx\Big |\\&\le \frac{1}{4N}\Vert g''\Vert _{L^1(I_j)} \end{aligned}$$

and, since $\lambda _j\in [\Lambda (j-\tfrac{1}{2}|{\mathfrak {q}}),\Lambda (j+\tfrac{1}{2}|{\mathfrak {q}})]$ thanks to $|{\text {Diff}}(j)|\le 1/2$,

$$\begin{aligned} \int _{\Lambda (j-1/2|{\mathfrak {q}})}^{\Lambda (j+1/2|{\mathfrak {q}})}|f(\lambda _j)-f(\Lambda (j|{\mathfrak {q}}))|\rho (\lambda |{\mathfrak {q}})d\lambda&=\frac{1}{N}|f(\lambda _j)-f(\Lambda (j|{\mathfrak {q}}))|\\&\le \frac{|{\text {Diff}}(j)|}{N}\Vert g'\Vert _{L^\infty (I_j)}\\&\le \frac{|{\text {Diff}}(j)|}{N}(\Vert g'\Vert _{L^1(I_j)}+\Vert g''\Vert _{L^1(I_j)}). \end{aligned}$$

Summing these estimates on *j* gives

$$\begin{aligned} \Big |\frac{1}{N} \sum _{j = 1}^n f(\lambda _j) - \int _{-{\mathfrak {q}}}^{{\mathfrak {q}}} f(\lambda ) \rho (\lambda | {\mathfrak {q}})\, d\lambda \Big | \le \frac{\mathrm {Diff}}{N}\Vert g'\Vert _{L^1([1/2,n+1/2])}+\frac{\tfrac{1}{4}+\mathrm {Diff}}{N}\Vert g''\Vert _{L^1([1/2,n+1/2])}. \end{aligned}$$

To conclude, observe that

$$\begin{aligned} \Vert g'\Vert _{L^1([1/2,n+1/2])}=\Vert f'\Vert _{L^1([-{\mathfrak {q}},{\mathfrak {q}}])} \end{aligned}$$

and

$$\begin{aligned} \Vert g''\Vert _{L^1([1/2,n+1/2])}\le \Big \Vert \frac{1}{N\rho (\cdot |{\mathfrak {q}})}\Big \Vert _{L^\infty ([-{\mathfrak {q}},{\mathfrak {q}}])}\Vert f''\Vert _{L^1([-{\mathfrak {q}},{\mathfrak {q}}])} +\Big \Vert \frac{\rho '(\cdot |{\mathfrak {q}})}{N\rho (\cdot |{\mathfrak {q}})^2}\Big \Vert _{L^\infty ([-{\mathfrak {q}},{\mathfrak {q}}])}\Vert f'\Vert _{L^1([-{\mathfrak {q}},{\mathfrak {q}}])} \end{aligned}$$

which, combined with the bounds

$$\begin{aligned} \zeta N\rho (x|{\mathfrak {q}}) \ge c_0(N-2n-C_0) \end{aligned}$$

(which is obtained from (46), the monotonicity of $\rho$ on $(-\infty ,0]$ and the assumption $\mathrm {Diff}\le 1/2$ which implies interlacement of $\pmb \lambda$ with $k = 1$) and $|\rho '(x|{\mathfrak {q}})|\le \tfrac{C_1}{\zeta }\rho (x|{\mathfrak {q}})$ (Proposition 24(ii)) on $[-{\mathfrak {q}},{\mathfrak {q}}]$, gives the claim. $\square$

### Claim 2

There exists a constant $C_2 > \frac{\pi }{2}C_0$ such that for every $\Delta \in (-1,1)$ and every $n\le N/2-C_2/\zeta$,

$$\begin{aligned} {\text {Diff}}\le \frac{C_2}{\zeta (N-2n-C_0)} \, , \end{aligned}$$

with $C_0$ being the constant arising in Claim 1.

### Proof

The constant $C_2$ will be chosen at the end of the proof; it will be apparent that it is independent of *n* or *N*. For $\Delta =0$, the result is obvious as the explicit (and unique) solution of the discrete Bethe Equation satisfies $\mathrm {Diff}=0$.

Assume that there exists $\Delta \in (-1,1)$ such that ${\text {Diff}}= \frac{C_2}{\zeta (N-2n)}\le \tfrac{1}{2}$. Using (33) in the first equality and then (19) in the second, we find

$$\begin{aligned} {\text {Diff}}(i)&=\frac{N}{2\pi }{\mathfrak {p}}(\lambda _i)-\int _{-{\mathfrak {q}}}^{\mathfrak {q}} \vartheta (\lambda _i-\mu )\rho (\mu |{\mathfrak {q}})d\mu -I_i\\&= \frac{1}{2\pi }\sum _{j=1}^n \vartheta (\lambda _i-\lambda _j) - \frac{N}{2\pi } \int _{-{\mathfrak {q}}}^{\mathfrak {q}} \vartheta (\lambda _i-\mu )\rho (\mu |{\mathfrak {q}})\, d\mu . \end{aligned}$$

Now, we use that for $K=\tfrac{1}{2\pi }\vartheta '$, $|K'|\le \tfrac{C}{\zeta }|K|$ and $\Vert K\Vert _{L^1[{\mathbb {R}}]}= 1-\tfrac{2\zeta }{\pi }$ (see the “Appendix” again). Apply Claim 1 to $\tfrac{1}{2\pi }\vartheta (\lambda _i-x)$ (and bound the $L^1$ norm on $[-{\mathfrak {q}},{\mathfrak {q}}]$ by the $L^1$ norm on ${\mathbb {R}}$) to get

$$\begin{aligned} |{\text {Diff}}(i)|&\le \mathrm {Diff}\cdot \Vert K\Vert _{L^1({\mathbb {R}})}+\frac{C_0(1+\mathrm {Diff})}{N-2n-C_0}(\zeta \Vert K'\Vert _{L^1({\mathbb {R}})}+\Vert K\Vert _{L^1({\mathbb {R}})}),\\&\le (1-\tfrac{2\zeta }{\pi })\mathrm {Diff}+\frac{C_0'}{N-2n-C_0}, \end{aligned}$$

where $C_0$ is the constant given by Claim 1, and $C_0'$ depends on $C_0$, but not on $C_2$. Since this applies to all *i*, we conclude that

67 $$\begin{aligned} \mathrm {Diff}\le \frac{\pi C_0'}{2\zeta (N-2n-C_0)}. \end{aligned}$$

Choose now $C_2$ so that $C_2 > \frac{\pi }{2} C_0'$. Then (67) contradicts our assumption on $\mathrm {Diff}$, and we conclude that there exists no $\Delta \in (-1,1)$ with ${\text {Diff}}= \frac{C_2}{\zeta (N-2n-C_0)}$. By the continuity of ${\text {Diff}}$ as a function of $\Delta$ and considering the fact that ${\text {Diff}}= 0$ for $\Delta = 0$, we conclude that ${\text {Diff}}< \frac{C_2}{\zeta (N-2n-C_0)}$ for all $\Delta \in (-1,1)$. $\square$

We are now in a position to conclude the proof of Theorem 22. Let $C = C_2$ be given by Claim 2 and fix *a*, *b*, *c* as in the theorem. By taking *N* large enough, we may assume that the value $\Delta$ corresponding to (*a*, *b*, *c*) is contained in the domain in which $\pmb \lambda$ is defined for any $n \le N/2 - C/\zeta$ (see Theorem 4). Then the dominant Eigenvalue may be expressed as

$$\begin{aligned} \tfrac{1}{N}\log \Lambda _N^{(n)}(\theta )=\ln a \, + \, \tfrac{1}{N}\sum _{j=1}^n {\mathscr {L}}(\lambda _j)+O(e^{-cN}), \end{aligned}$$

where ${\mathscr {L}}(\cdot )$ is the function defined in (58). Claims 1 and 2 give

$$\begin{aligned}&\Big |\tfrac{1}{N}\log \Lambda _N^{(n)}-\int _{-{\mathfrak {q}}}^{{\mathfrak {q}}} {\mathscr {L}}(\lambda )\rho (\lambda |{\mathfrak {q}})d\lambda \Big |\\&\quad \le \frac{C_3}{\zeta N(N-2n-C_0)}\Vert {\mathscr {L}}'\Vert _{L^1({\mathbb {R}})}+\frac{C_3}{\zeta N(N-2n-C_0)}(\zeta \Vert {\mathscr {L}}''\Vert _{L^1({\mathbb {R}})}+\Vert {\mathscr {L}}'\Vert _{L^1({\mathbb {R}})})\\&\quad \le \frac{C_0}{\zeta N(N-2n-C_0)}. \end{aligned}$$

Furthermore, Sects. 7.1 and 7.2 give that

$$\begin{aligned} \int _{-{\mathfrak {q}}}^{{\mathfrak {q}}} {\mathscr {L}}(\lambda )\rho (\lambda )d\lambda =f(a,b,c) - C(\Delta )(1+o(1))\sin \theta (1-\tfrac{2n}{N})^2. \end{aligned}$$

The above implies (66) by choosing *C* large enough. $\square$

## A Formulae for the Different Functions and Their Fourier Transforms

Recall the parameterisations (2), (3) and ( 4) of the weights *a*, *b*, *c*. We remind that we always assume that $a\ge b>0$, which corresponds to $\theta \in (0, \pi /2]$.

### A.1 Case $|\Delta |<1$

If $\zeta :=\arccos (-\Delta )\in (0, \pi )$, we have for $x \in {\mathbb {R}}$, using the principal branch of the logarithm,

$$\begin{aligned} {\mathfrak {p}}(x)&:= i \log {\displaystyle \frac{\sinh {(i \zeta / 2 + x)}}{\sinh {(i \zeta / 2 - x)}}} \, = \, 2\arctan [\tanh (x)\cot (\zeta /2)],\\ \vartheta (x)&:=i \log {\displaystyle \frac{\sinh {(i \zeta + x)}}{\sinh {(i \zeta - x)}}} \, = \, 2 \arctan \big [ \tanh (x) \cot (\zeta )\big ],\\ K(x)&:=\tfrac{1}{2\pi }\vartheta '(x) =\frac{1}{\pi }\frac{\sin {(2\zeta )}}{\cosh {(2x)} - \cos {(2\zeta )}},\\ \xi (x)&:=\tfrac{1}{2\pi }{\mathfrak {p}}'(x) =\frac{1}{\pi }\frac{\sin {(\zeta )}}{\cosh {(2x)} - \cos {(\zeta )}},\\ \rho (x)&:=\frac{1}{2\zeta \cosh {(\pi x / \zeta )}},\\ {\mathscr {L}}(x)&:=\tfrac{1}{2}\log [L(x)L(-x)]. \end{aligned}$$

Moreover, the following direct consequences of the formulas above are used in the text: ${\mathfrak {p}}$ is strictly increasing, odd and ${\mathfrak {p}}({\mathbb {R}}) = (-\pi + \zeta /2,\pi -\zeta /2)$; $\vartheta$ is decreasing for $\Delta > 0$, increasing for $\Delta < 0$ and constant for $\Delta = 0$; and finally $\vartheta ({\mathbb {R}}) =(- |\pi -\zeta |, |\pi -\zeta |)$.

We will also use the following Fourier transforms (with the relevant continuous extension at $t=0$ when needed):

$$\begin{aligned} \widehat{K}(t)&= \frac{\sinh [(\pi - 2 \zeta ) t/2]}{\sinh {[\pi t/2]}} ,\\ \widehat{\xi }(t)&= \frac{\sinh [(\pi - \zeta ) t/2]}{\sinh {[\pi t/2]}}, \\ \widehat{\rho }(t)&= \frac{1}{2\cosh {(\zeta t/2)}},\\ \widehat{ {\mathscr {L}}}(t)&= \frac{2\pi \sinh ( \tfrac{ \theta }{\pi } \zeta t) }{ t } \widehat{\xi }(t). \end{aligned}$$

### A.2 Case $\Delta = -1$

We have

$$\begin{aligned} {\mathfrak {p}}(x)&:= i \log {\displaystyle \left( \frac{i / 2 + x}{i / 2 - x}\right) },\\ \vartheta (x)&:=i \log {\displaystyle \left( \frac{i + x}{i - x}\right) },\\ K(x)&:=\tfrac{1}{2\pi }\mathfrak \vartheta '(x) = \frac{1}{\pi (1+x^2)},\\ \xi (x)&:=\tfrac{1}{2\pi }{\mathfrak {p}}'(x)=\frac{2}{\pi (1+4x^2)},\\ \rho (x)&:= \frac{1}{2\cosh {[\pi x]}},\\ {\mathscr {L}}(x)&:=\tfrac{1}{2}\log [L(x)L(-x)] = \tfrac{1}{2}\log \Big [\frac{x^2+\left( \frac{1}{2}+\frac{\theta }{\pi }\right) ^2}{x^2+\left( \frac{1}{2} - \frac{\theta }{\pi }\right) ^2}\Big ]. \end{aligned}$$

We will also be interested in the following Fourier coefficients (with relevant extensions at $t=0$)

$$\begin{aligned} \widehat{K}(t)&=e^{-|t|}, \\ \widehat{\xi }(t)&=e^{-|t|/2}, \\ \widehat{\rho }(t)&=\frac{\widehat{\xi }(t)}{1+\widehat{K}(t)} = \frac{1}{2\cosh {[t/2]}},\\ \widehat{ {\mathscr {L}} }(t)&=\pi \cdot \frac{e^{-\frac{a-b}{2c}\cdot |t|}\cdot (1-e^{-|t|})}{|t|} . \end{aligned}$$

### A.3 Case $\Delta < -1$

Recall that $\zeta = \mathrm{arccosh}( -\Delta ) >0$. We have

$$\begin{aligned} K(x)&:=\tfrac{1}{2\pi }\vartheta '(x) =\frac{1}{\pi }\frac{\sinh {(2\zeta )}}{\cosh {(2\zeta )} - \cos {(2x)}},\\ \xi (x)&:=\tfrac{1}{2\pi }{\mathfrak {p}}'(x) =\frac{1}{\pi }\frac{\sinh {(\zeta )}}{\cosh {(\zeta )} - \cos {(2x)}},\\ \rho (x)&:= \tfrac{1}{2\pi } \sum _{n\in {\mathbb {Z}}}\frac{e^{2inx}}{\cosh {[n\zeta ]}}=\tfrac{1}{2\zeta }\sum _{n\in {\mathbb {Z}}}\frac{1}{\cosh {[\pi (\pi n - x)/\zeta ]}},\\ {\mathscr {L}}(x)&:=\tfrac{1}{2}\log [L(x)L(-x)]. \end{aligned}$$

The functions ${\mathfrak {p}}$ and $\vartheta$ are then defined as the odd smooth functions on ${\mathbb {R}}$ that have $\tfrac{1}{2\pi }\xi$ and $\tfrac{1}{2\pi }K$ as derivatives. In particular, on $(-\tfrac{\pi }{2},\tfrac{\pi }{2})$, they are equal to

$$\begin{aligned} {\mathfrak {p}}(x)&:= i \ln {\displaystyle \frac{\sin (i \zeta /2 + x)}{\sin (i \zeta /2 - x)}},\\ \vartheta (x)&:=i \ln {\displaystyle \frac{\sin (i \zeta + x)}{\sin (i \zeta - x)}}. \end{aligned}$$

Moreover, the following direct consequences of the formulas above are used in the text: ${\mathfrak {p}}$ is increasing and maps ${\mathbb {R}}$ to ${\mathbb {R}}$; $\vartheta$ is increasing; $\vartheta ([-\pi /2,\pi /2]) =[-\pi , \pi ]$ and $\vartheta$ extends to ${\mathbb {R}}$ as a quasi-periodic continuous function. The function *K* is even, unimodal, and has zero limits at $\pm \infty$.

We stress that these formulae do not extend, *per se*, beyond $(-\tfrac{\pi }{2},\tfrac{\pi }{2})$. We will also be interested in the following Fourier coefficients, when $2\theta /\pi <1$,

$$\begin{aligned} \widehat{K}(n)&=e^{-2|n|\zeta }, \\ \widehat{\xi }(n)&=e^{-|n|\zeta }, \\ \widehat{\rho }(n)&=\frac{\widehat{\xi }(n)}{1+\widehat{K}(n)} = \frac{1}{2\cosh {[n\zeta ]}},\\ \widehat{ {\mathscr {L}}}(n)&=\frac{\pi }{2|n|}(e^{-|n|\cdot \left| 1-\frac{2\theta }{\pi }\right| \zeta } - e^{-|n|(1+\frac{2\theta }{\pi })\zeta }) =\frac{\pi }{n}\mathrm {e}^{-|n|\zeta } \sinh \big [2 \zeta n \tfrac{\theta }{\pi } \big ] \end{aligned}$$

if $$n\ne 0$$, and $$\widehat{ {\mathscr {L}}}(0)=2\theta \zeta $$.

### A.4 Case $\Delta =-\infty$

We have

$$\begin{aligned} {\mathfrak {p}}(x)&:=2x,\\ \vartheta (x)&:=2x,\\ K(x)&:=\tfrac{1}{\pi },\\ \xi (x)&:=\tfrac{1}{\pi },\\ \rho (x)&:= \tfrac{1}{2\pi }. \end{aligned}$$

## B Analysis of Continuum Bethe Equation for $|\Delta |<1$

In this appendix we gather some information on $\rho (x|q)$ when $|\Delta |<1$. The first proposition justifies the existence of this quantity.

### Proposition 23

(Existence of solutions to (15)). For every $|\Delta |<1$ and $q\ge 0$, there exists a unique solution $x\mapsto \rho (x|q)$ to (15). Furthermore, for every $m\in [0,1/2]$, there exists *Q*(*m*) satisfying (16).

### Proof

Direct computation shows that the operator ${\mathcal {K}}$ on $L^1([-q,q])\cap L^\infty ([-q,q])$ defined by

$$\begin{aligned} {\mathcal {K}}[f](x):=\int _{-q}^q K(x-y)f(y)dy \end{aligned}$$

satisfies

68 $$\begin{aligned} \Vert {\mathcal {K}} \Vert _{L^\infty \rightarrow L^\infty } \le \Vert {\mathcal {K}} \Vert _{L^1 \rightarrow L^1} = \Vert K \Vert _{L^1({\mathbb {R}}) } = |\vartheta (+\infty )-\vartheta (-\infty )|= \frac{|4 \zeta - 2\pi |}{2\pi } < 1.\nonumber \\ \end{aligned}$$

It follows that ${\text {Id}}+ {\mathcal {K}}$ is invertible and the solution $\rho (\lambda | q)$ is unique and lies in $L^1({\mathbb {R}}) \cap L^\infty ({\mathbb {R}})$ with a uniform bound on the norm. Since *K*(*x*) is smooth in *x* and *q*, the Fredholm series representation for the resolvent of ${\text {Id}}+ {\mathcal {K}}$ [23] allows one to infer that $(x,q,\Delta )\mapsto \rho (x | q)$ is smooth.

The existence of *Q*(*m*) follows readily from the continuity of the map $(x, q,\Delta )\mapsto \rho (x|q)$, the mean-value theorem, and the fact that $\rho (x|0)$ integrates to 0 while $\rho (\cdot )=\rho (\cdot |Q(\tfrac{1}{2}))$ to $\tfrac{1}{2}$. $\square$

The following proposition gives the necessary properties for the proof of Theorem 4 when $-1< \Delta <0$ (see Sect. 3.2).

### Proposition 24

(Properties necessary for Theorem 4). Then there exist $c,C>0$ such that for every $-1 < \Delta \le 0$,

(i): For $$q\in {\mathbb {R}}$$ and $$x\in {\mathbb {R}}$$, $$0<\rho (x)\le \rho (x|q)\le \rho (x)+\rho (q)$$.
(ii): For $q\in {\mathbb {R}}$ and $x\in {\mathbb {R}}$, $|\rho '(x|q)| \le \frac{C}{\zeta }(\rho (x|q)+\rho (q))$.
(iii): For every $m\in {\mathbb {R}}$, $\tfrac{c}{\zeta }(\tfrac{1}{2}-m)\le \rho (Q(m))\le \tfrac{C}{\zeta }(\tfrac{1}{2}-m)$.

The lower bound of (i) was first established in [12].

### Proof

Recall that ${\widehat{R}}=\widehat{K}/(1+\widehat{K})$. Following [42], one gets that $R\ge 0$ since ${\widehat{R}}= {\widehat{\rho }}\widehat{K}/ \widehat{\xi }$ and therefore

69 $$\begin{aligned} R(\lambda ) = \int \limits _{ {\mathbb {R}} }{} \rho (\lambda -y)F(y)dy \end{aligned}$$

in which $\rho$ is obviously positive while

$$\begin{aligned} F(x) = \frac{1}{2\pi } \int \limits _{ {\mathbb {R}} }{} \frac{{\widehat{K}}(\omega )}{ {\widehat{\xi }}(\omega )} \mathrm {e}^{i x \omega }d \omega \, = \frac{1}{\pi -\zeta }\frac{ \sin {(\frac{\pi \zeta }{\pi -\zeta } )} }{ \cosh {(\frac{2 \pi x}{\pi -\zeta })} - \cos {(\frac{\pi \zeta }{\pi -\zeta } )} } > 0 \;, \end{aligned}$$

where the second equality follows from a straightforward residue computation.

The operator ${\mathcal {U}}$ on $L^\infty ({\mathbb {R}})\cap L^1({\mathbb {R}})$ defined by

$$\begin{aligned} {\mathcal {U}}[f](x):=\int _{{\mathbb {R}}{\setminus }[-q,q]}R(x-y)f(y)dy \end{aligned}$$

satisfies

70 $$\begin{aligned}&\max \big \{ \Vert {\mathcal {U}} \Vert _{L^\infty ({\mathbb {R}}) \rightarrow L^\infty ({\mathbb {R}})} \, , \, \Vert {\mathcal {U}} \Vert _{L^1({\mathbb {R}}) \rightarrow L^1({\mathbb {R}})} \big \} \le \Vert R\Vert _{L^1({\mathbb {R}})}\nonumber \\&\quad ={\widehat{R}}(0)= \frac{\widehat{K}(0)}{1 + \widehat{K}(0)} =\frac{\pi - 2 \zeta }{2 \pi - 2 \zeta } < \frac{1}{2} \end{aligned}$$

so that the version (23) of (15) immediately gives that

71 $$\begin{aligned} \rho (x|q)&=\sum _{k=0}^{\infty }{\mathcal {U}}^k[\rho ](x). \end{aligned}$$

This expression and the fact that $R\ge 0$ gives the lower bound of (i). For the upper bound, we isolate the first term in the sum and then use operator bounds to get that

$$\begin{aligned} \rho (x|q)&\le \rho (x)+\sum _{k=1}^\infty \Vert {\mathcal {U}}^k\Vert _{L^\infty ({\mathbb {R}})\rightarrow L^\infty ({\mathbb {R}})}\cdot \Vert \rho \,\pmb {\mathbb {1}}_{|x|>q}\Vert _\infty \le \rho (x)+\rho (q). \end{aligned}$$

To prove (ii), differentiate (23) with respect to $\lambda$ and then integrate by parts to obtain that $\rho '(\cdot |q)$ satisfies the functional equation:

$$\begin{aligned} \rho '(x|q)-\int _{ {\mathbb {R}} {\setminus } [-q,q] }  R(x-y)\rho '(y|q)\, dy = \rho '(x)+[R(x-q)-R(x+q)]\rho (q|q). \end{aligned}$$

In particular

$$\begin{aligned} | \rho '(x|q) | \le |\rho '(x)| + 2\Vert R \Vert _\infty \rho (q| q) \le |\rho '(x)| + 4\tfrac{{\widetilde{C}}}{\zeta } \rho (q), \end{aligned}$$

since $\Vert R \Vert _\infty < {\widetilde{C}} / \zeta$ and $\rho (q| q)< 2\rho (q)$. Finally, using $|\rho '(x)|\le \tfrac{\pi }{\zeta }\rho (x)$ we obtain the desired bound for a well-chosen value of *C*.

We now focus on (iii). The definition of *m*, (23), and

$$\begin{aligned} \widehat{R}(0) \, = \, \frac{{\widehat{K}}(0)}{1+{\widehat{K}}(0)} \, = \, \frac{\pi - 2 \zeta }{2 \pi - 2 \zeta }<\frac{1}{2} \end{aligned}$$

give that

72 $$\begin{aligned} \tfrac{1}{2}-m&=\int \limits _{{\mathbb {R}}}\rho (x)dx-\int \limits _{-Q(m)}^{Q(m)}\rho (x|Q(m))dx\nonumber \\&=  \int \limits _{ [-Q(m),Q(m)]^{\mathrm {c}} }  \rho (x|Q(m))dx -  \int \limits _{ {\mathbb {R}} \times [-Q(m),Q(m)]^{\mathrm {c}} }  R(x-y)\rho (y|Q(m)) dy dx\nonumber \\&=\tfrac{ \pi }{2 \pi - 2 \zeta }  \int \limits _{ [-Q(m),Q(m)]^{\mathrm {c}} }  \rho (x|Q(m))dx \; > \; \tfrac{ \pi }{2 \pi - 2 \zeta }  \int \limits _{ [-Q(m),Q(m)]^{\mathrm {c}} }  \rho (x)dx\nonumber \\&= (1 + o(1)) \; \tfrac{ 2 \zeta }{2 \pi - 2 \zeta } \rho (Q(m)), \end{aligned}$$

as $m\rightarrow 1/2$ (since then $Q(m)\rightarrow + \infty$). Above, we used the notation $[-Q(m),Q(m)]^{\mathrm {c}} := {\mathbb {R}}{\setminus }[-Q(m),Q(m)]$. Plugging again the expression (71) in this estimate to replace $\rho (\cdot |Q(m))$ by $\rho$ in the integral, and then using the explicit formula for $\rho$ gives the result easily. $\square$

We finish with the properties necessary to obtain Theorem 2 for $|\Delta |<1$.

### Proposition 25

(Properties necessary for Theorem 2). There exists $C>0$ such that for every $|\Delta |<1$:

(i): There exists a unique solution $T\in \big ( L^{\infty }\cap L^1\big )({\mathbb {R}})$ of the functional equation 73 $$\begin{aligned} T(x) -\int \limits _0^\infty R(x-y)T(y)dy = {\mathfrak {e}}(x) \quad \text {with} \quad {\mathfrak {e}}(x) \, := \, \tfrac{1}{\zeta } \mathrm {e}^{-x \frac{\pi }{\zeta }} \pmb {\mathbb {1}}_{{\mathbb {R}}_+}(x). \end{aligned}$$
(ii): For every $q\ge 0$ and $x\in {\mathbb {R}}$, 74 $$\begin{aligned} \rho (x|q)=\rho (x) + \mathrm {e}^{-q \frac{\pi }{\zeta } } \big [ (T-{\mathfrak {e}})(q-x) + (T-{\mathfrak {e}})(-q-x) + \delta T(x) \big ] \, , \end{aligned}$$ where $\Vert \delta T\Vert _\infty +\Vert \delta T\Vert _1\le Ce^{-2q}$.
(iii): It holds $$\begin{aligned}\displaystyle \lim _{m\rightarrow 1/2} ( \tfrac{1}{2}-m) \mathrm {e}^{Q(m) \frac{\pi }{\zeta } } \;=\; \frac{ \pi }{ \pi - \zeta } \int \limits \limits _{0}^{+\infty } T(\lambda )d \lambda . \end{aligned}$$

Note that, in fact, one may solve (73) in terms of a scalar Riemann–Hilbert problem by implementing the Wiener-Hopf method. However, we will not need such a precise information on *T* and will thus establish Proposition 25 by more elementary means.

### Proof

We stress that, below, all domination relations *O*(*f*) will be uniform in $\Delta$, *viz*.  bounded by *Cf* with *C* being $\Delta$ independent. We start with Item (i). Introduce the operator ${\mathcal {V}}$ on $L^\infty ({\mathbb {R}})\cap L^1({\mathbb {R}})$ defined by

$$\begin{aligned} {\mathcal {V}}[f](x):=\int \limits _{{\mathbb {R}}_+}R(x-y)f(y)dy. \end{aligned}$$

which has $\Vert \cdot \Vert _{L^1({\mathbb {R}})\rightarrow L^1({\mathbb {R}})}$-operator norm smaller than 1/2, owing to the chain of bounds $\Vert {\mathcal {V}}[f] \Vert _1 \, \le \, \Vert R \Vert _\infty \Vert f\Vert _1 \, \le \, \tfrac{1}{2} \Vert f \Vert _1$. This justifies the existence and uniqueness of $T\in L^1({\mathbb {R}})$ and gives the formula

75 $$\begin{aligned} T= \sum _{k=0}^\infty {\mathcal {V}}^k[ {\mathfrak {e}}] . \end{aligned}$$

For item (ii), introduce the operators

$$\begin{aligned} {\mathcal {U}}_+ [f](x)&:= {\mathcal {V}}[f( q+\cdot )](-q + x) \; = \; \int \limits _{q}^{+\infty }R(x-y)f(y) d y \\ {\mathcal {U}}_- [f](x)&:= {\mathcal {V}}[f(- q-\cdot )](-q - x) \; = \; \int \limits ^{-q}_{-\infty }R(x-y)f(y) d y \end{aligned}$$

and observe that ${\mathcal {U}} [f] = {\mathcal {U}}_+[f] + {\mathcal {U}}_-[f]$. Further, by introducing the operators $\tau _{\pm }, \check{\tau }_{\pm }$ such that $\tau _{\pm }[f](x)=f(\pm q \pm x)$ and $\check{\tau }_{\pm }[f](x)=f(- q \pm x)$, one finds that ${\mathcal {U}}_\pm = \check{\tau }_{\pm } {\mathcal {V}} \tau _{\pm }$. Therefore, one gets that

$$\begin{aligned} \rho (\lambda |q)&= \rho (\lambda ) \, + \, \sum _{ k \ge 1}^{} ( {\mathcal {U}}_+ + {\mathcal {U}}_-)^k[\rho ](\lambda ) \, = \, \rho (\lambda ) \, + \, \sum _{ k \ge 1}^{}( {\mathcal {U}}_{+} ^k + {\mathcal {U}}_{-} ^k) [\rho ] \, + \, \delta T_{\text {pert}}(\lambda ) \, , \end{aligned}$$

in which

76 $$\begin{aligned} \delta T_{\text {pert}}(\lambda )\, := \, \sum _{ k \ge 1} \sum _{p=1}^{k-1} \sum _{ \begin{array}{c} \epsilon _i \in \{ \pm \} \\ \# \{ i: \epsilon _i=+ \} = p \end{array} }^{}  \big ( {\mathcal {U}}_{\epsilon _1} \cdots {\mathcal {U}}_{\epsilon _k} \big ) [\rho ](\lambda ) . \end{aligned}$$

A direct calculation yields

77 $$\begin{aligned} {\mathcal {U}}_+{\mathcal {U}}_-[f](\lambda ) = \int \limits _{0}^{+\infty } R(-q+\lambda - \mu )d \mu \int \limits _{-\infty }^{-q} R(q+\mu -\nu ) f(\nu )d \nu . \end{aligned}$$

In order to estimate the norm of ${\mathcal {U}}^+{\mathcal {U}}^-$ one recalls the convolution representation (69) for *R* which shows that *R* is decreasing on ${\mathbb {R}}_+$ and enjoys the bound

78 $$\begin{aligned} R(\lambda ) \, = \, O( {\mathfrak {b}}(\lambda ) ) \quad \text {as} \quad \lambda \rightarrow + \infty \quad \text {and} \; \text {where} \quad {\mathfrak {b}}(\lambda ) := \max \big \{ \mathrm {e}^{ -\frac{2\pi }{\pi -\zeta } |\lambda | }, \mathrm {e}^{ -\frac{\pi }{\zeta } |\lambda | } \big \} .\;\;\qquad \end{aligned}$$

This immediately yields

$$\begin{aligned} \Vert {\mathcal {U}}_+{\mathcal {U}}_-[f] \Vert _{ L^{1}({\mathbb {R}}) } \, \le \, C \, {\mathfrak {b}}(2q) \, \Vert R \Vert _{ L^{1}({\mathbb {R}}) } \Vert f \Vert _{ L^{1}({\mathbb {R}}) }, \end{aligned}$$

for some constant *C*. Clearly, similar bounds do hold for $\Vert {\mathcal {U}}_-{\mathcal {U}}_+[f] \Vert _{ L^{1}({\mathbb {R}}) }$.

Now, observe that $\check{\tau }_{\pm }\tau _{\pm }= {\text {Id}}$, so that $\big ( {\mathcal {U}}_{\pm } \big )^k[\rho ] = \check{\tau }_{\pm } {\mathcal {V}}^{k}[\rho (\pm q \pm \cdot )]$. The identity

79 $$\begin{aligned} \rho (\pm q \pm x) \, = \, \mathrm {e}^{-\frac{\pi }{\zeta }q} {\mathfrak {e}}(x) \Big ( 1 + \delta {\mathfrak {e}}(x) \Big )\qquad \text {with} \qquad \delta {\mathfrak {e}}(x) := \frac{ - \mathrm {e}^{-2\frac{\pi }{\zeta }(q+x) } }{ 1+ \mathrm {e}^{-2\frac{\pi }{\zeta }(q+x) } } \;, \end{aligned}$$

which holds uniformly for $x \ge 0$ yields

80 $$\begin{aligned} \sum _{ k \ge 1}^{} \big ( {\mathcal {U}}_{\pm } \big )^k [\rho ] \, = \, \mathrm {e}^{-\frac{\pi }{\zeta }q} \check{\tau }_{\pm }\big [ T - {\mathfrak {e}}\big ] \; + \; \mathrm {e}^{-\frac{\pi }{\zeta }q} \delta T_{\pm } \quad \text {with} \quad \delta T_{\pm } \, = \, \sum _{ k \ge 1} \check{\tau }_{\pm } \cdot {\mathcal {V}} ^k [ {\mathfrak {e}} \delta {\mathfrak {e}} ]\nonumber \\ \end{aligned}$$

By using that

81 $$\begin{aligned} \Vert {\mathcal {U}}_{\pm } \Vert _{ L^{1/\infty }({\mathbb {R}} ) \rightarrow L^{1/\infty }({\mathbb {R}}) } \, = \, \Vert {\mathcal {V}} \Vert _{ L^{1/\infty }({\mathbb {R}}) }< \varkappa _\zeta = \frac{ \pi -2 \zeta }{2 ( \pi - \zeta ) }< \tfrac{1}{2}, \end{aligned}$$

one infers that

$$\begin{aligned} \Vert \delta T_{\pm } \Vert _{ L^{1/\infty }({\mathbb {R}}) } \, \le \, \sum _{k \ge 1}^{} \frac{1}{2^k} \Vert {\mathfrak {e}} \delta {\mathfrak {e}} \Vert _{ L^{1/\infty }({\mathbb {R}}_+) } \, \le \, C \mathrm {e}^{- \frac{2\pi }{\zeta } q } . \end{aligned}$$

Further, given $\epsilon _i\in \{\pm \}$ such that $\# \{ i \, : \, \epsilon _i = + \}\in \{1,\dots , k-1\}$, there is necessarily at least one change of sign in the string $\epsilon _1,\dots , \epsilon _k$ so that one gets

$$\begin{aligned} \Vert {\mathcal {U}}_{\epsilon _1} \cdots {\mathcal {U}}_{\epsilon _k} [\rho ] \Vert _{ L^{1/\infty }({\mathbb {R}}) }&= \varkappa _{\zeta }^{k-2} \cdot \text {e}^{-\frac{\pi }{\zeta } q}\cdot \max \Big \{ \Vert {\mathcal {U}}_{+} {\mathcal {U}}_{-} \Vert _{ L^{1/\infty }({\mathbb {R}}) } , \Vert {\mathcal {U}}_{-} {\mathcal {U}}_{+} \Vert _{ L^{1/\infty }({\mathbb {R}}) } \Big \} \\&\le C \cdot \varkappa _{\zeta }^{k-2} \cdot \text {e}^{-\frac{\pi }{\zeta } q}\cdot {\mathfrak {b}}(2q) . \end{aligned}$$

Above, $\mathrm {e}^{- \frac{ \pi }{\zeta } q }$ issues from the estimates of the action of ${\mathcal {U}}^\pm$ on $\rho$. This leads to the estimate on $\delta T_{\text {pert}}$ introduced in (76):

$$\begin{aligned} \Vert \delta T_{\text {pert}} \Vert _{ L^{1/\infty }({\mathbb {R}}) }\,&\le \, C \cdot \sum _{ k \ge 1 }^{} \sum _{p=1}^{k-1} C_{k}^{p} \varkappa _{\zeta }^{k-2} {\mathfrak {b}}(2q) \cdot \mathrm {e}^{- \frac{ \pi }{\zeta } q } \,\\&\le \, C \mathrm {e}^{- \frac{ \pi }{\zeta } q } {\mathfrak {b}}(2q) \cdot \sum _{ k \ge 1 }^{} (2 \varkappa _{\zeta })^{k}= C^{\prime }(\zeta ) \mathrm {e}^{- \frac{ \pi }{\zeta } q } {\mathfrak {b}}(2q) . \end{aligned}$$

Thus, one obtains the representation (74) with $\delta T$ given by

82 $$\begin{aligned} \delta T = \delta T_+ + \delta T_- + \delta T_{\text {pert}} \quad \text {and} \quad \Vert \delta T \Vert _{ L^{1/\infty }({\mathbb {R}}) }= O\big ( {\mathfrak {b}}(2q) + \mathrm {e}^{- 2 \frac{ \pi }{\zeta } q } \big ). \end{aligned}$$

For Item (iii), recall the exact representation for $\tfrac{1}{2} - m$ given in (72). Then, by virtue of (74) and upon using the symmetry of the operator *T*, one gets

83 $$\begin{aligned} \int \limits _{ [-q,q]^{\text {c}} }^{ }  \rho (\lambda |q) d \lambda \,= & {} \, 2\int \limits _{q}^{\infty } \rho (\lambda ) d \lambda \, +\,2 \mathrm {e}^{- \frac{ \pi }{\zeta } q } \int \limits _{0}^{ \infty } \big ( T - {\mathfrak {e}}\big ) (\lambda ) d \lambda \, \nonumber \\&+ \, 2 \mathrm {e}^{- \frac{ \pi }{\zeta } q } \int \limits _{q}^{\infty } \big ( T(-q-\lambda ) + \delta T(\lambda ) \big ) d \lambda . \end{aligned}$$

Here, we used that ${\mathfrak {e}}$ has support on ${\mathbb {R}}_{+}$. The part involving $\delta T$ can be directly estimated when $q \rightarrow + \infty$ to give $O({\mathfrak {b}}(2q)+\mathrm {e}^{-2\frac{\pi }{\zeta } q})$ Using the explicit expression for $\rho (\lambda )$, it is easy to see that

$$\begin{aligned} \int \limits _{q}^{\infty } \rho (\lambda ) d \lambda = (1 + o(1)) \, \mathrm {e}^{- \frac{ \pi }{\zeta } q } \int \limits _{0}^{ \infty } {\mathfrak {e}} (\lambda ) d \lambda . \end{aligned}$$

Further, by using the integral equation satisfied by T, one gets that

$$\begin{aligned} \int \limits _{q}^{\infty } T(-q-\lambda ) d \lambda \,&= \, \int \limits _{-\infty }^{-2q} T(\lambda )d \lambda \, = \, \int \limits _{-\infty }^{0} \int \limits _{0}^{\infty } R(\lambda -2q - \mu ) T(\mu ) d\mu d\lambda \\&\le \Vert T\Vert _{L^{1}({\mathbb {R}}) } \int \limits _{0}^{\infty } R(2q+ \lambda )d\lambda \, \le \, C {\mathfrak {b}}(2q) . \end{aligned}$$

Thus, by substituting *Q*(*m*) in place of *q* in the above estimates and using that $Q(m)\rightarrow + \infty$ as $m\rightarrow \tfrac{1}{2}$ by virtue of Item (iii) of Proposition 24, one obtains that

$$\begin{aligned} \tfrac{1}{2} - m \, = \, \mathrm {e}^{ - \frac{ \pi }{\zeta } Q(m) } \frac{ \pi }{ \pi - \zeta } \bigg \{ \int \limits _{0}^{ \infty } T (\lambda ) d \lambda \, + \, O\Big ( {\mathfrak {b}}( 2 Q(m) ) \, + \, \mathrm {e}^{- 2 \frac{ \pi }{\zeta } Q(m) } \Big ) \bigg \} . \end{aligned}$$

Therefore

$$\begin{aligned} \lim _{m \rightarrow \frac{1}{2} } \big \{ \tfrac{1}{2} - m \big \}\mathrm {e}^{ \frac{ \pi }{\zeta } Q(m) } \; = \; \frac{ \pi }{ \pi - \zeta } \int \limits _{0}^{ \infty } T (\lambda ) d \lambda . \end{aligned}$$

The constant on the right-hand side is strictly positive since *T* is given by a sum of strictly positive terms. $\square$

## C Analysis of Continuum Bethe Equation When $${\Delta }$$ = $$ -1$$

### Proposition 26

(Existence of solutions to (15)). Fix $\Delta = -1$ and $q\ge 0$, there exists a unique solution $x\mapsto \rho (x|q)$ to (15). Furthermore, for every $m\in [0,1/2]$, there exists *Q*(*m*) satisfying (16).

### Proof

The proof valid for $|\Delta |<1$ does not generalise directly since estimating that the operator ${\mathcal {K}}$ has $\Vert \cdot \Vert _{L^1\rightarrow L^1}$-norm strictly less than 1 demands more effort, see [28]. However, by working with (23) instead of (15), one readily checks that the fact that the operator ${\mathcal {U}}$ has norm smaller than $\tfrac{1}{2}$, which gives the existence of solutions. The rest of the proof is the same. $\square$

The following proposition gives the necessary properties for the proof of Theorem 2 when $\Delta = -1$. The proof is the same as for $|\Delta |<1$.

### Proposition 27

Fix $\Delta = -1$. There exists $C>0$ such that

(i): There exists a unique solution $T\in L^1({\mathbb {R}})$ of the functional equation 84 $$\begin{aligned} T(x) -\int \limits _0^\infty R_{-1}(x-y)T(y)dy = {\mathfrak {e}}(x) \equiv \mathrm {e}^{- x \pi } \pmb {\mathbb {1}}_{{\mathbb {R}}_+}(x). \end{aligned}$$
(ii): We have that 85 $$\begin{aligned} \rho (x|q) \, =\, \rho (x) + \mathrm {e}^{-q\pi } \big [ (T-{\mathfrak {e}})(q-x)+(T-{\mathfrak {e}})(-q-x) + \delta T(x) \big ] \, , \end{aligned}$$ where $\Vert \delta T\Vert _{L^{\infty }({\mathbb {R}})}+\Vert \delta T\Vert _{L^{1}({\mathbb {R}})}\le Ce^{-2q}$.
(iii): $\displaystyle \lim _{m\rightarrow 1/2} ( \tfrac{1}{2}-m) \mathrm {e}^{ Q(m)\pi }$ exists and belongs to $(0,\infty )$.

Note that, above, $R_{-1}$ stands for the resolvent kernel to the operator $\mathrm{Id} + {\mathcal {K}}$ at $\Delta =-1$ and acting on $L^2({\mathbb {R}})$.

## D Computations for $\Delta < -1$

### Proposition 28

For every $\Delta < -1$ and $q\in [0,\pi /2]$, there exists a unique solution $x\mapsto \rho (x|q)$ to (15). Furthermore, there exists *Q*(*m*) satisfying (16).

### Proof

In order to circumvent the problems with estimating $\Vert {\mathcal {K}}\Vert _{ L^1([-q,q]) \rightarrow L^1([-q,q])}$, we again work with the alternative representation of the integral equation (23) in which

$$\begin{aligned} R(\lambda ) \, =\, \frac{1}{2\pi } \sum \limits _{ n \in {\mathbb {Z}} }{} \frac{ \mathrm {e}^{2i \lambda - |n|\zeta } }{ \cosh (n \zeta ) } . \end{aligned}$$

Then, it remains to use that $\Vert R \Vert _{L^{1}([-\pi /2,\pi /2]}=1/2$ so as to conclude as in the $\Delta =-1$. Finally, the existence of *Q*(*m*) follows from the same argument as in the $|\Delta |<1$ case. $\square$

### Lemma 29

It holds $$\rho (x|q)\ge \rho (x)\ge \frac{1}{2\zeta }$$

### Proof

The explicit expression for $\rho$ implies that $\rho (x)\ge \frac{1}{2\zeta }$. It is thus enough to establish the upper bound. This follows from an expression similar to (71) for $\Delta <-1$ and the fact that $R\ge 0$. To check the latter, note that

$$\begin{aligned} {\widehat{R}}(n)={\widehat{\rho }}(n)\frac{{\widehat{K}}(n)}{{\widehat{\xi }}(n)}={\widehat{\rho }}(n){\widehat{\xi }}(n)=\frac{e^{-2|n|\zeta }}{1+e^{-2|n|\zeta }}\end{aligned}$$

so that *R* is the convolution of $\rho$ and $\xi$ which are both positive. $\square$

### Lemma 30

For $\Delta <-1$, we get

$$\begin{aligned} \lim _{m\rightarrow 1/2}\frac{1-2m}{\pi -2 Q (m) } = \rho ( \tfrac{\pi }{2} ). \end{aligned}$$

### Proof

Equation (23) and the observation that $\Vert R\Vert _{L^1([-\frac{\pi }{2}, \frac{\pi }{2}])}=\tfrac{1}{2}$ along with the continuity of $\rho$ give

$$\begin{aligned} \tfrac{1}{2}-m&=\int \limits _{-\frac{\pi }{2} }^{ \frac{\pi }{2} }\rho (x)dx-\int \limits _{-Q (m) }^{ Q (m) }\rho (x| Q (m) )dx \\&= \big ( \pi -2Q (m) \big )\rho (\tfrac{\pi }{2})[1+o(1)] - \int \limits _{-Q (m)}^{Q (m)} \int \limits _{ [-Q (m), Q (m)]^{\mathrm {c}} }  \rho (y|Q (m))R(x-y)\, dy\, dx. \\&= \big ( \tfrac{\pi }{2} - Q (m) \big ) \rho (\tfrac{\pi }{2})[1+o(1)]. \end{aligned}$$

where we set $[-Q (m), Q (m)]^{\mathrm {c}}: = [-\pi /2, \pi /2]{\setminus } [-Q (m), Q (m)]$. $\square$

## Footnotes

1 The existence and uniqueness of *r*, $\zeta$ and $\theta$ is proved via basic computations. Notice that the parameter *r* has no influence on the probabilistic behaviour of the model.

2 This was, however, achieved on a formal level in [9].

3 The absolute value in $\log |L(\lambda )|$ is harmless, as the symmetry of $\pmb \lambda$ implies that $\prod _{j=1}^n L(\lambda _j)$ is real and positive.

4 When $|\Delta |\le 1$, we consider square-integrable functions on ${\mathbb {R}}$. For *F* in $L^2({\mathbb {R}})$, the Fourier transform of *F* is given by

$$\begin{aligned}{\widehat{F}}(t):=\int _\mathbb Re^{-itx}F(x)dx.\end{aligned}$$

When $\Delta <-1$, we consider $\pi$-periodic functions that are square integrable on $[-\pi /2,\pi /2]$ (call $L_\pi ^2({\mathbb {R}})$ the set of $\pi$-periodic functions *f* with $\int _0^\pi |f(t)|^2dt<\infty$). Then, for $f\in L^2_\pi ({\mathbb {R}})$, define the Fourier coefficients $\widehat{f}:{\mathbb {Z}}\rightarrow {\mathbb {C}}$ by

$$\begin{aligned} f(t)=\frac{1}{\pi }\sum _{n\in {\mathbb {Z}}} \widehat{f}(n) e^{2int}. \end{aligned}$$

5 Meaning that $A_{ii} > \sum _{j\ne i} | A_{ij} |$ for every *i*.

6 We also use the trivial facts that $K(0)\ge 0$, $\rho (\lambda | {\mathfrak {q}})\ge 0$ and $K=\tfrac{1}{2\pi }\vartheta '$ in the first inequality.

7 The displayed inequality can be obtained from the easy observation (already made in a number of papers, see e.g. [18]) that there exists a map from configurations with $n+1$ up arrows per row to configurations with *n* up arrows per row constructed by choosing a path of oriented edges cycling around the torus in the vertical direction with length smaller than *MN*/*n* (such a path always exists and there are at most $N4^{MN/n}$ choices for it) and reversing all arrows on it. This changes the weights, hence the factor $\min \{a,b,c\}/\max \{a,b,c\}$.
